# The Infectious Disease Ontology in the age of COVID-19

**DOI:** 10.1186/s13326-021-00245-1

**Published:** 2021-07-18

**Authors:** Shane Babcock, John Beverley, Lindsay G. Cowell, Barry Smith

**Affiliations:** 1 Department of Philosophy, Niagara University, Lewiston, NY USA; 2 National Center for Ontological Research, University at Buffalo, Buffalo, NY USA; 3 Department of Philosophy, Northwestern University, Evanston, IL USA; 4 Cowell Lab, University of Texas Southwestern Medical Center, Dallas, TX USA; 5 Department of Philosophy, University at Buffalo, Buffalo, NY USA

**Keywords:** Coronavirus, COVID-19, Infectious disease, Infectious disease ontology, Ontology, Data integration

## Abstract

**Background:**

Effective response to public health emergencies, such as we are now experiencing with COVID-19, requires data sharing across multiple disciplines and data systems. Ontologies offer a powerful data sharing tool, and this holds especially for those ontologies built on the design principles of the Open Biomedical Ontologies Foundry. These principles are exemplified by the Infectious Disease Ontology (IDO), a suite of interoperable ontology modules aiming to provide coverage of all aspects of the infectious disease domain. At its center is IDO Core, a disease- and pathogen-neutral ontology covering just those types of entities and relations that are relevant to infectious diseases generally. IDO Core is extended by disease and pathogen-specific ontology modules.

**Results:**

To assist the integration and analysis of COVID-19 data, and viral infectious disease data more generally, we have recently developed three new IDO extensions: IDO Virus (VIDO); the Coronavirus Infectious Disease Ontology (CIDO); and an extension of CIDO focusing on COVID-19 (IDO-COVID-19). Reflecting the fact that viruses lack cellular parts, we have introduced into IDO Core the term *acellular structure* to cover viruses and other acellular entities studied by virologists. We now distinguish between *infectious agents* – organisms with an infectious disposition – and *infectious structures* – acellular structures with an infectious disposition. This in turn has led to various updates and refinements of IDO Core’s content. We believe that our work on VIDO, CIDO, and IDO-COVID-19 can serve as a model for yielding greater conformance with ontology building best practices.

**Conclusions:**

IDO provides a simple recipe for building new pathogen-specific ontologies in a way that allows data about novel diseases to be easily compared, along multiple dimensions, with data represented by existing disease ontologies. The IDO strategy, moreover, supports ontology coordination, providing a powerful method of data integration and sharing that allows physicians, researchers, and public health organizations to respond rapidly and efficiently to current and future public health crises.

**Supplementary Information:**

The online version contains supplementary material available at 10.1186/s13326-021-00245-1.

## Background

Efforts by physicians, researchers, and public health organizations to respond to infectious diseases require the use of multiple, constantly changing data sources. Consider, for instance, a research team trying to model a given population’s herd immunity to measles. This depends on the integration of data not merely from biology and medicine, but also from public health, geography, and social science [1]. Because such data is collected using discipline- and community-specific methodologies and is stored in geographically distributed and often non-interoperable databases, the data are typically only locally accessible. The resultant silo-formation [2] hinders both translational and comparative research and preventive and prognostic public health research [3]. These problems can be solved by traditional means with the investment of sufficient time and effort. In circumstances of public health emergency, however, more powerful methods for data sharing and integration must be applied.

As the experience of biologists and bioinformaticians has shown, ontologies – logically well-designed, structured, vocabularies – are a powerful data sharing tool [4]. But to be effective, ontologies must be designed in a coordinated fashion – otherwise ontologies themselves will give rise to the creation of a new kind of silo [2]. One of the most successful and widely adopted approaches to coordinated ontology development is that of the Open Biomedical Ontologies (OBO) Foundry [5], a collective of developer groups dedicated to creating, testing, and maintaining a suite of ontologies based on an evolving set of ontology design principles:

- Ontologies should use a well-specified syntax and share a common space of identifiers.
- Ontologies should be openly available in the public domain for reuse.
- Ontologies in neighboring domains should be developed in a collaborative effort.
- Ontologies should be developed in a modular fashion.
- Ontologies should have a clearly specified scope.
- Ontologies should use common unambiguously defined relations between their terms.
- Ontologies should conform to a common top-level architecture.

The OBO Foundry principles were modelled initially on the practices of the Gene Ontology (GO) [4], which has served as the model for subsequent life science ontologies [6].

Wherever possible, OBO ontologies are created using terms, relational expressions, and definitions taken from existing OBO ontologies, including the Relation Ontology (RO) [7], which ensures cross-linkage between ontologies in neighboring domains and also helps avert redundant efforts. Basic Formal Ontology (BFO) is the official top-level ontology for all OBO Foundry ontologies. BFO, which is comprised of highly general classes such as ‘object’, ‘material entity’, and ‘process’, is used by more than 350 ontology projects as their top-level architecture [2] and has been approved as international standard ISO/IEC 21838–2 [8, 9]. Ontology construction and extension in accordance with OBO principles follows a ‘hub and spokes’ model, where a core or ‘hub’ ontology provides the basis for extension ontologies providing domain-specific terms at progressively lower levels. The Infectious Disease Ontology (IDO), first released in 2010 [10], was constructed in this manner, and consequently, provides a central ‘hub’ from which ‘spoke’ ontologies extend to more specific disease domains.

### IDO Core, BFO and OGMS

IDO Core covers just those entities that are relevant to infectious diseases generally, and not to specific infectious diseases associated with specific pathogens. Its coverage ranges across biological scales (gene, cell, organ, organism, population), disciplinary perspectives (biological, clinical, epidemiological), and successive stages along the chain of infection (host, reservoir, vector, pathogen) [11]. At the heart of IDO Core is the term ‘disease’, which is imported from the Ontology for General Medical Science (OGMS) [12]. Developers of OGMS view the traditional practice of classifying diseases according to patterns of similarities in signs and symptoms as inadequate. A single disease may manifest a variety of symptoms, making it difficult to distinguish the disease definitionally from other diseases involving the same anatomical system [13]. To address such issues, OGMS characterizes diseases in BFO terms as *dispositions* of patients to undergo pathological processes of specific kinds. Distinguishing manifestations of symptoms from dispositions to manifest symptoms provides the flexibility needed to represent diseases that have multiple different sorts of presentation [12]. The OGMS approach allows, moreover, for the existence of pre-clinical manifestations of disease, and for clinical risk factor combinations of disease and predispositions to disease (as when AIDS in a given patient is a risk factor for a second disease such as tuberculosis [14]). Supplementary Table 1 and Additional File 1 detail ontologies which make use of the OGMS approach to disease. Table 1 below reflects OGMS definitions relevant to our discussion of IDO Core.

**Table 1:** Definitions imported from OGMS to IDO Core

OGMS Term	Definition
*disease*	Disposition to undergo pathogenic processes that exists in an organism because of one or more disorders in that organism.
*disease course*	Totality of all processes through which a given disease instance is realized.
*disorder*	Material entity which is clinically abnormal and part of an extended organism; disorders are the physical basis of disease.
*symptom*	Process experienced by the patient, which can only be experienced by the patient, that is hypothesized to be clinically relevant.

The term *disease* relies on *organism* in its definition. The class *organism* is imported from the Ontology for Biomedical Investigations (OBI) [15] and defined as “an object that is an individual living system, such as animal, plant, bacteria, or virus, that is capable of replicating or reproducing, growth and maintenance in the right environment. An organism may be unicellular or made up, like humans, of many billions of cells divided into specialized tissues and organs.” (As we shall see below, the reference to ‘virus’ here is problematic. We are working with the OBI developers to address this matter [16].) The term *disorder* relies on the class *extended organism* and elucidation of “clinically abnormal” in its definition. Instances of *extended organism* are object aggregates consisting of an organism and all material entities located within that organism overlapping the organism or occupying sites formed in part by the organism. *Extended organism* comprises not only the organism itself but also the normal microflora and invading pathogens contained within it and the pathogens on its surface, as well as the sites, for example the oral or nasal cavities, which these pathogens may occupy. Clinical abnormality is a feature of an organism that is not part of the life plan for an organism of the relevant type (unlike aging or pregnancy), is causally linked to an elevated risk either of pain or other feelings of illness, or of death or dysfunction, and is such that the elevated risk exceeds a certain threshold level [12].

## Results

Recent development of three IDO extension ontologies – the Virus Infectious Disease Ontology, the Coronavirus Infectious Disease Ontology, and IDO-COVID-19 – has proceeded concurrently with updates and refinements to IDO Core’s existing content, as well as new term imports from related OBO Ontologies. In the following we  detail a selection of the relevant updates. We then detail the development of VIDO, CIDO and IDO-COVID-19.

### Extending IDO Core from OGMS

**Fig. 1:**
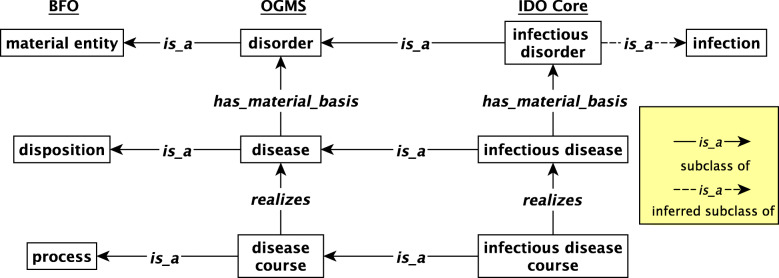
Relationships between disease, disorder, and disease courses in IDO Core

The IDO Core extension of relevant classes from OGMS is represented visually in Fig. 1 and with textual definitions in Table 2. Subclasses of entities are linked by BFO relations such as *realizes* and *has_material_basis*, the latter used to indicate the material basis of a disposition, in this case, a disease.

**Table 2:** IDO Core definitions extending from OGMS

IDO Core Term	Definition
*infection*	Material entity part of an extended organism that has some pathogen as part, which participates in the formation of the material entity by invading tissues of the organism.
*infectious disorder*	Disorder that is part of an extended organism which has an infectious pathogen part, that exists as a result of a process of formation of disorder initiated by the infectious pathogen.
*infectious disease*	Disease whose physical basis is an infectious disorder.
*infectious disease course*	Disease course that is the realization of an infectious disease.

#### Pathogens and infectious entities

The term *infection* relies on *pathogen*, which is defined in IDO Core as a material entity bearing a pathogenic disposition. The term *infectious disorder* relies on *infectious pathogen*, which IDO Core defines as a pathogen bearing an infectious disposition. Corresponding updates in the most recent version of IDO Core are illustrated in Table 3.

**Table 3:** IDO Core definitions of infectious pathogens

IDO Core Term	Definition
*acellular structure*	Object consisting of an arrangement of interrelated acellular parts forming an acellular biological unit that is able to initiate replication of the structure in a host.
*pathogenic disposition*	Disposition borne by a material entity to establish localization in or produce toxins that can be transmitted to an organism or acellular structure, either of which may form a disorder in the entity or in immunocompetent members of the entity’s species.
*infectious disposition*	Pathogenic disposition borne by a pathogen to be transmitted to a host and then become part of an infection in that host or in immunocompetent members of the same species as the host.
*establishment of localization in host*	Process in which a material entity reaches a site in or on a host in which it can survive, grow, multiply, or mature and establishes itself there.
*process of establishing an infection*	Process by which an infectious agent or infectious structure, established in a host, becomes part of an infection in the host.
*appearance of disorder*	Process through which a disorder comes into existence.

Motivation for these updates stems from reflection on the fact that importing the term *organism* from OBI implies viruses fall under this class and are cellular entities. Viruses are, however, acellular. To avoid this issue, IDO Core introduces the term *acellular structure* instances of which lack cellular parts, as a parent class for the class *virus* which is imported from NCBITaxon [17]. IDO Core now distinguishes between *infectious agents* – organisms bearing an infectious disposition – and *infectious structures* – acellular structures bearing an infectious disposition. The use of “organism or acellular structure” in the definitions of pathogenic and infectious dispositions reflects, moreover, the fact that viruses themselves may be the targets of infection by, say, other viruses.

The definition of *pathogenic disposition* refers to the ability of a pathogen to participate in an *establishment of localization in host*, which can often form a disorder if the subsequent maturing and/or multiplying of the pathogen in the host leads to tissue damage. The definition’s first disjunctive clause covers cases where pathogens cause a disorder without ever localizing in a host, as when foodborne toxins produced by *clostridium botulinum* are ingested, leading to food botulism. The definition also covers cases involving both mechanisms, as when the intestines of infants are colonized by *C. botulinum* and secreted toxins are then absorbed into the bloodstream.

The definition’s second disjunctive clause allows for cases of localization that do not lead to disorders but are nevertheless contagious. An example would be the localization of HIV-1 in a human host that is resistant to the virus due to a mutation of the CCR-5 gene that blocks the virus from attaching to host cells, and so blocks pathogenesis to AIDS [18]. The virus’s presence is not clinically abnormal as it is not causally linked to an elevated risk of either pain or other feelings of illness, or of death or dysfunction in the resistant host. But while the virus is unable to fully realize its infectious disposition in the host, it is still disposed to transmit to and bring clinical abnormality to other potential immunocompetent human hosts without the mutation.^1^

In making *infectious disposition* a child of *pathogenic disposition*, IDO Core distinguishes pathogenicity and infectiousness. *C. botulinum*, for example, is a pathogen, but not infectious. After an infant ingests honey colonized by *C. botulinum*, the bacterium secretes toxins into the bloodstream, resulting in disorder. But it does not itself become part of that disorder, nor is it disposed to be transmitted to new potential hosts. And so *infant botulism* is non-infectious. By contrast, COVID-19 is infectious precisely because it is rooted in an infectious disorder composed of SARS-CoV-2 viruses disposed to be transmitted to other potential hosts.

**Fig. 2:**
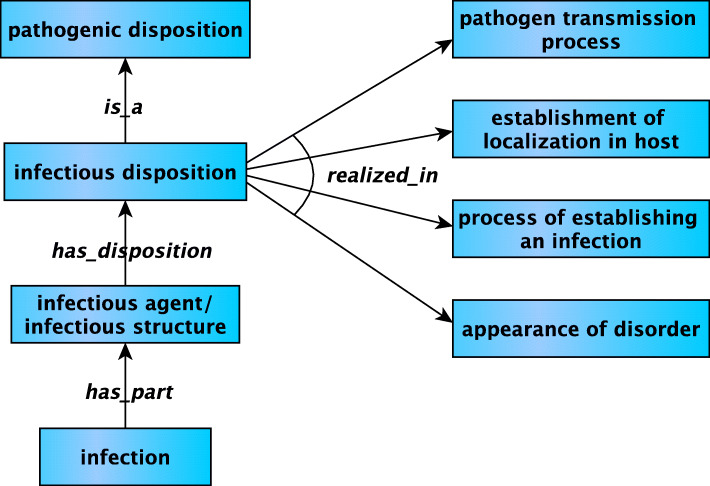
Some aspects of IDO *infectious disposition*

We have throughout made reference to “hosts” of pathogens and infectious entities. In IDO Core instances of *host* bear a *host role*, which is a role borne by an acellular structure containing a distinct material entity, or organism whose extended organism contains a distinct material entity, realized in use of that structure or organism as a site of reproduction or replication. Reference to *acellular structure* accommodates the case of a virus that serves as host to an infecting virophage. Our definition also provides the resources needed to characterize the implicit temporal ordering in the definition of *infectious disposition* represented in Fig. 2.

When an infectious entity realizes its infectious disposition, it is first transmitted to the host before establishing localization in the host, after which it will become part of an infection prior to the appearance of disorder. If the virus establishes itself in the host, becomes part of an infection—*and* that infection is causally linked to an elevated risk of either pain or other feelings of illness, or of death or dysfunction in the host—then the infection is also an *infectious disorder*, and so the host is disposed to undergo various pathological processes. But infection may also occur without such clinical abnormality, in which case the virus has failed to fully realize its infectious disposition, and so has failed to establish an *infectious disorder*.

Before turning to pathogen transmission in more detail, two points are worth making here. First, our definitions do not count the presence of commensal microflora in our microbiome, many of which bear an *infectious disposition*, as constituting either an *infection* or an *infectious disorder*. This is because the microbiome part of our extended organism is not formed by an invasive *process of establishing an infection*. Here we have only *colonization* of the host. Under normal circumstances the relevant pathogens are unable to realize their infectious disposition. Yet they can still form disorders in their hosts, if they end up in the wrong anatomical site, as in the case of bacteremia, or if a colony grows out of control, as in the case of yeast infections. Each of these cases involves an *opportunistic pathogen*, defined in IDO Core as a pathogen with an *opportunistic infectious disposition*, in turn defined as an infectious disposition to become part of a disorder only in organisms whose defenses are compromised.^2^ Thus our definitions allow for the representation of infectious disorders caused by organisms that are typically commensal.

**Fig. 3:**
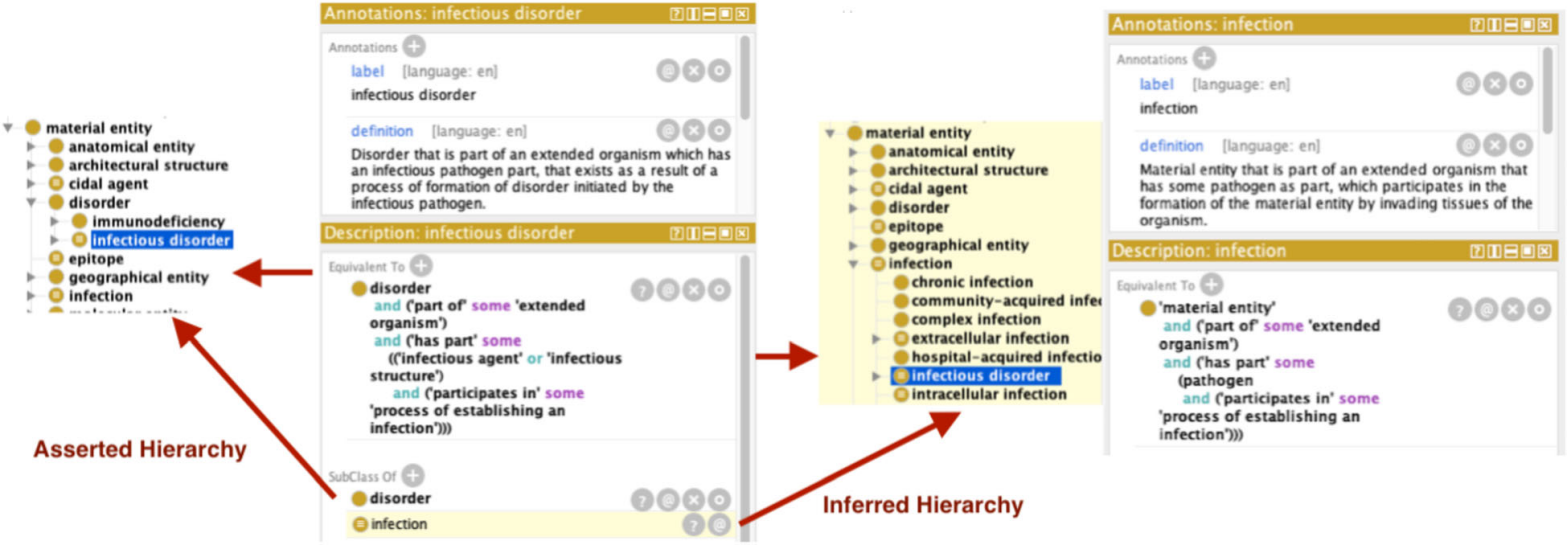
Infectious Disorder inferred subclass of infection

Second, the preceding definitions are accompanied by logical axioms used in querying and automated reasoning over the ontology. Figure 3, for example, illustrates how the axioms relating to *infectious disorder* entail, rightly, that it is an inferred subclass of *infection*.

#### Transmission of pathogens and infectious entities

IDO Core characterizes infectious disease transmission in its various forms. From the Pathogen Transmission Ontology [19] IDO Core imports terms such as^3^*pathogen transmission process*, which is a process during which a pathogen is transmitted directly or indirectly to a new host, and *indirect pathogen transmission process*, which is a pathogen transmission process in which a pathogen is indirectly transferred to a host by intermediary vehicles or vectors. Infectious diseases vary widely in associated transmission processes. For example, malaria and dengue fever are vector borne, while Schistosoma helminth parasites spend part of their life cycle within intermediate hosts after which they are transmitted into another medium, such as water, which then directly transmits the pathogen to definitive hosts such as humans. Table 4 reports IDO Core definitions relevant to a wide range of transmission types.

**Table 4:** IDO Core transmission definitions

IDO Core Term	Definition
*pathogen transporter role*	Role borne by a material entity in or on which a pathogen is located, from which the pathogen may be transmitted to a new host.
*pathogen vector role*	Pathogen transporter role that is borne by an organism active in the transfer of an infectious agent or infectious structure to an organism of another species in which it can realize its infectious disposition.
*host role*	Role borne by an acellular structure containing a distinct material entity, or organism whose extended organism contains a distinct material entity, realized in use of that structure or organism as a site of reproduction or replication.
*pathogen host role*	Host role borne by an organism having a pathogen as part of its extended organism.
*symbiont host role*	Host role borne by an organism whose extended organism provides an environment supportive for the survival, growth, maturation, or reproduction of an object contained as a proper part.

While the definition of *pathogen transporter role* requires the bearer to actually have a pathogen located in or on it, bearers also have certain dispositions that enable them to play this role. While a mosquito bears the *pathogen vector role* only when a malaria parasite is located in or on it, there also inheres in its physical structure a *disposition* to transfer the parasites, which it has whether or not it contains any parasites. Similarly, for respiratory droplets that serve as vehicles for viruses such as SARS CoV-2. Notice also that a mosquito plays the vector role even when it is actively transferring a malaria parasite to a non-infectable human being bearing the sickle-cell trait. What is important is that the parasite is being transferred to an organism of a *species* in which its infectious disposition can typically be realized. A mosquito is not playing the vector role when transferring the parasite to an organism of a non-susceptible species.

The preceding selection does not exhaust those host roles included in IDO Core but does reflect the wide range of ways in which to characterize host-symbiont relationships.

#### Pathogen inhibition and control

IDO Core provides terms relevant to treatment agents, such as *cidal agent* and *static agent*. The former is the bearer of a *cidal agent disposition*, a disposition realized in the killing of bacteria, fungi, parasites, or viruses. The latter is the bearer of a *static agent disposition*, a disposition realized in a process of inhibiting the reproduction of bacteria, fungi, or parasites, or a process of inhibiting the replication of viruses. Subclasses of *cidal agent disposition*, such as *bactericidal disposition* (disposition to kill bacteria) and *viricidal disposition* (disposition to kill viruses), as well as *cidal agent* subclasses—such as *bactericidal* and *viricide*—are defined in the corresponding pathogen-type IDO reference ontologies (see below ). The same for pathogen-type subclasses of *static agent disposition* and *static agent*. Agents that target only specific bacterial, fungal, parasitic, or viral species are to be defined in IDO extensions for the specific pathogens.

By our definitions the immune system, and the cells and cellular entities that constitute it, bear both cidal and static agent dispositions (as do devices such as autoclaves and sterilizers). Many drugs work not by directly killing or inhibiting pathogens, but rather by ramping up the immune system. While many associate terms like *bactericidal* and *viricide* with drugs and other chemical substances, researchers also use such terms to describe proteins in the immune system, especially interferon-gamma which is secreted by T helper cells.

Related is another notable aspect of IDO, which is its treatment of the phenomenon of *resistance* [20]. Examples include a population’s herd immunity to certain infectious agents and the resistance of certain pathogens to antimicrobial drugs. IDO Core characterizes this phenomenon as involving an entity bearing *protective resistance*, a disposition that inheres in the entity by virtue of it having some part which is disposed to mitigate damage to the entity. For instance, a host’s immunity to a given virus is a type of *protective resistance*. The host has certain parts, such as immune cells, that are disposed to secrete antibodies, neutralizing viral particles, and preventing the virus from infecting the host. *Protective resistance* is further characterized in terms of a “blocking disposition” [11, 20], a disposition the manifestation of which prevents, or mitigates, the realization of another disposition. Thus, the disposition of a host’s immune cell acts as a blocking disposition since the process of antibody secretion prevents the virus from realizing its own disposition to infect and cause damage to the host.

We have refined the definition of *protective resistance* to narrow its scope, now defining it as a “Disposition inhering in an acellular structure or organism, with a part having a disposition to mitigate damage to the entity from internal and invasive threats, which is realized in one or more negative biological regulation processes.” The last clause refers to the GO class *negative regulation of biological process*, a process that stops, prevents, or reduces the frequency, rate or extent of a biological process. Thus, my blocking of a knife thrust is not the realization of a *protective resistance*, as a knife thrust is not a biological process. When, in contrast, a virus evades a host immune response (a biological process) it is realizing a *protective resistance*. A related case study is provided in Additional File 3**.**

#### Epidemiology and surveillance

**Fig. 4:**
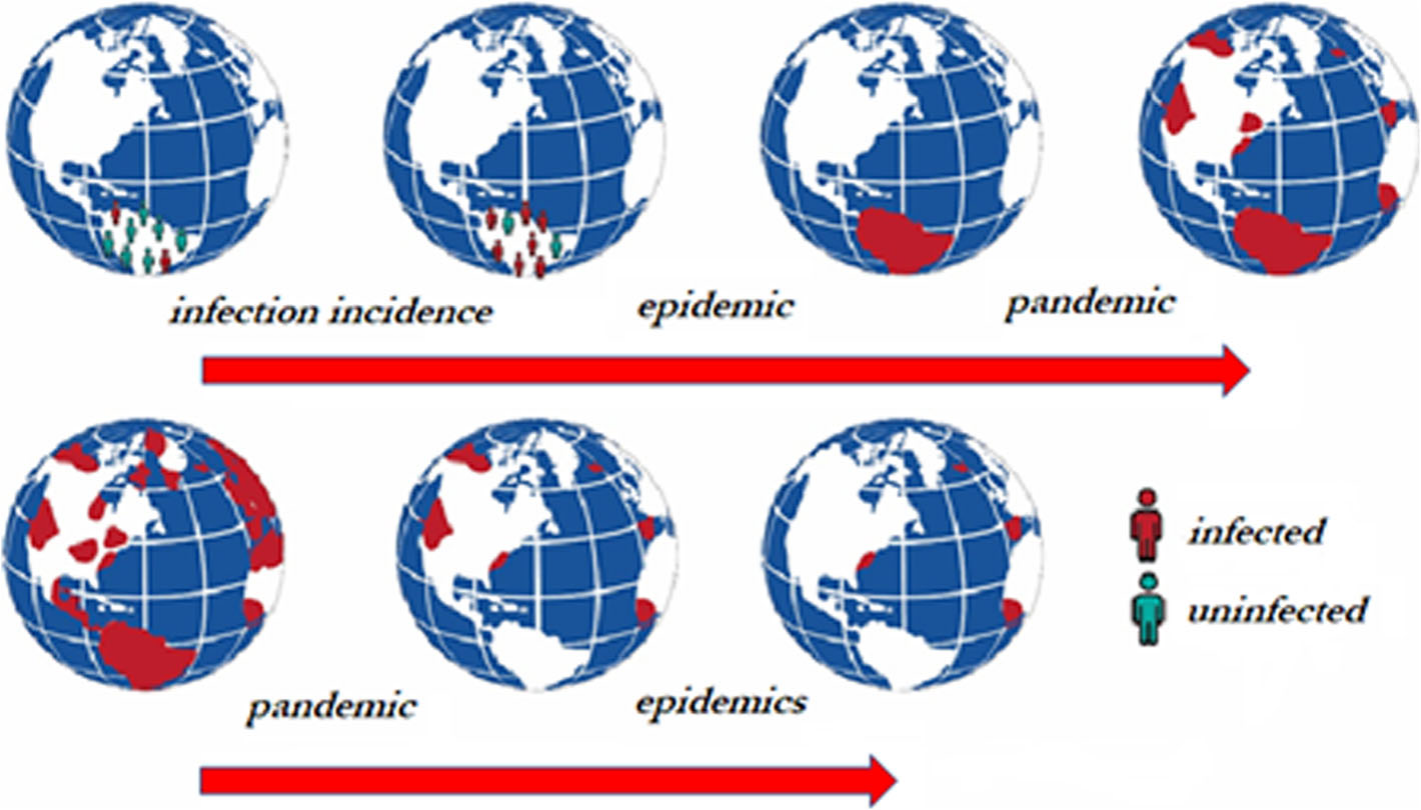
Transitions through epidemic and pandemic

IDO Core includes terms for population-level processes, such as the epidemiological spread of disease as represented in Fig. 4.

When an infection incidence in a population increases beyond a certain threshold in a geographic region, this may signal an epidemic in the region. When epidemics emerge in distinct geographic regions, this may signal the emergence of a pandemic. Over time, a pandemic may involve more or fewer geographic regions, and remain a pandemic. However, once the number of epidemics decreases below a certain threshold, there is no longer a pandemic. Similarly, the distribution of infections among members of a population may change while sustaining an epidemic, but once the infection incidence falls below a certain threshold, there is no longer an epidemic. IDO Core terms in Table 5 provide resources needed to represent these phenomena.

**Table 5:** IDO Core epidemiological terms

IDO Core Term	Definition
*infectious disease epidemic*	Process of infectious disease realizations for which there is a statistically significant increase in the infectious disease incidence of a population.
*infectious disease pandemic*	Process in which multiple infectious disease epidemics of the same type of infectious disease unfold over overlapping periods of time and affect organism populations located in different geographic regions, including different countries and continents.
*infectious disease incidence*	Quality that inheres in an organism population and is the number of realizations of an infectious disease for which the infectious disease course begins during a specified period of time.
*directive information content entity (IAO)*	Information Content Entity that consists of a set of propositions or images (as in the case of a blueprint) that prescribes some Entity.
*disease surveillance objective specification (APOLLO-SV)*	Objective specification whose endpoint is human awareness of the level of a disease in a particular population of a given biological taxon during some time interval.
*infectious disease control objective specification (APOLLO-SV)*	Objective specification that is realized by processes that are able or likely to stop the spread of a disease in a population.
*infectious disease control strategy* *(APOLLO-SV)*	Plan specification whose objective specification is an infectious disease control objective specification.
*contact tracing (APOLLO-SV)*	Infectious disease control strategy that identifies and treats contacted organisms in a host population.
*quarantine control strategy (APOLLO-SV)*	Infectious disease control strategy whereby asymptomatic carriers who have had contact with pathogens are prevented from having contact with other susceptible organisms.

In addition to *infectious disease incidence*, IDO Core includes other qualities of infected populations, such as *infectious disease mortality rate* and *infectious disease endemicity*. IDO Core’s coverage of epidemiology has been enhanced with a variety of term imports from the Apollo Structured Vocabulary (Apollo-SV) [21], which provides a standardized vocabulary for terms and relations required for the interoperation between epidemic simulator models and public health application software that interface with these models. Apollo-SV draws heavily on the Information Artifact Ontology (IAO) [22], and the terms in Table 5 which have been imported to IDO Core from Apollo-SV are subclasses of the IAO class *directive information content entity*. IDO Core has also been expanded with new classes, including *pathogen surveillance* and *vector surveillance*. The former are surveillance processes aiming to produce information about one or more pathogens with the purpose of managing those pathogens, while the latter are surveillance processes aiming to produce information about changes in the geographical distribution and density of one or several pathogen vectors with the purpose of facilitating appropriate and timely decisions regarding interventions.

### Extensions of IDO Core

IDO Core is a hub from which a variety of spoke ontologies covering specific infectious diseases extend. Table 6 provides a list of the IDO Core extensions at their current state of development. Details of these ontologies can be found within Additional File 1 in Supplementary Table 2 and Supplementary Table 4. Other disease ontologies employing IDO terms are discussed in Supplementary Table 3. Supplementary Table 5 and Supplementary Table 6 detail databases and other applications to which IDO Core and its extensions have been applied.

**Table 6:** IDO Extension Ontologies

*Coronavirus Infectious Disease Ontology (CIDO)* [23–25]	Most recent version uploaded to Bioportal on June 11, 2021 [26]
*Influenza Ontology (IDOFLU)* [27]	Most recent version uploaded to BioPortal on August 20, 2015 [28]
*Brucellosis Ontology (IDOBRU)* [29]	Most recent version uploaded to BioPortal on March 28, 2015 [30]
*IDO Virus (VIDO)* [31]	Most recent version uploaded to BioPortal on July 25, 2021 [32]
*COVID-19 Infectious Disease Ontology* (IDO-COVID-19) [31]	Most recent version uploaded to BioPortal on July 25, 2021 [33]
*Dengue Fever Ontology (IDODEN)* [34]	Most recent version uploaded to BioPortal on February 17, 2014 [35]
*Malaria Ontology (IDOMAL)* [36]	This ontology is obsoleted but is being hosted for legacy purposes [37].
*Meningitis Ontology (IDOMEN)* [38]	Draft version uploaded on November 27, 2019 [39]
*Plant Disease Ontology (IDOPlant)* [40]	Draft version released in 2012 [41]
*Staphylococcus aureus Infectious Disease Ontology (IDOSA)* [11, 19, 42]	Released on June 22, 2012 [43]
*Schistosomiasis Ontology (IDOSCHISTO)* [44]	Most recent version uploaded on October 23, 2013 [45]
*HIV Ontology (IDOHIV)* [46]	Most recent version uploaded to BioPortal on April 4, 2017 [47]

Ideally, all IDO Core extension ontologies would be developed in the same way, and in conformance to all Foundry principles. Unfortunately, not all of the Foundry principles have been followed faithfully by the IDO Core extension ontologies represented in Table 6. Surveying extensions of IDO Core revealed a range of issues in these extensions, which are detailed at length, alongside recommendations for correction, in Additional File 2.

#### Partitioning the IDO suite and creating a lattice of infectious disease ontologies

While currently existing IDO extensions were designed as direct extensions of IDO Core, several extension ontologies have defined terms which are not included in IDO Core, but which are useful where a group of extension ontologies cover the same pathogen type. For example, the term *virion* – a single complete virus particle – is needed for each IDO Core extension covering viral infectious diseases, but is not needed in, say, representations of fungal infectious diseases. These observations suggest the need for pathogen-type specific reference ontology extensions of IDO Core. IDO Core extensions can easily be partitioned into subgroups based on pathogen type. For example, CIDO and IDOFLU both cover infectious viral diseases while IDOBRU and IDOSA both cover infectious bacterial diseases.

Additionally, the range of issues identified in Additional File 2 provides motivation for coordinated partitioning of the IDO suite of ontologies. IDO Core extensions were often developed without sustained coordination with nearby extensions. While we discuss how we intend to address such coordination issues below, for our purposes here, we note that creating pathogen-type specific reference ontology extensions of IDO Core creates fewer opportunities for misalignment among extensions. Much like a researcher who seeks to represent influenza can rely on IDO Core as a reliable starting point, and so not need to reflect on what exactly, say, an “infectious disease” is, similarly the same researcher importing a virus-specific extension of IDO Core would not need to reflect on what exactly, say, a “virus” is.

**Fig. 5:**
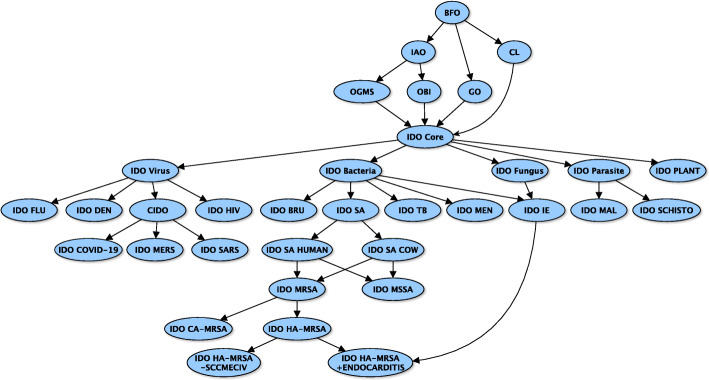
A lattice of infectious disease ontologies

Grouping IDO Core extensions based on pathogen type is coordinated by the development of reference ontologies comprised of terms common to scientific investigations of the relevant pathogen. The resulting ontologies themselves extend directly from IDO Core and provide a hub from which pathogen-specific ontologies extend. Partitioning IDO Core extensions based on pathogen type results in bacteria, virus, fungi, and parasite specific reference ontologies, as illustrated in Fig. 5. IDOSA, IDOMEN, IDOTB, IDOIE and IDOBRU extend from IDO Bacteria. IDOFLU, IDOHIV, IDODEN and CIDO extend from IDO Virus, while IDOSCHISTO and a new ontology for malaria (replacing IDOMAL) extend from IDO Parasite.

[42] shows how IDOSA annotations of genetic, phenotypic, and demographic data on *S. aureus* isolates maintained by the Network on Antimicrobial Resistance in *Staphylococcus aureus* [48] can be used to infer lattice application ontologies for specific subfamilies of *S. aureus*-related diseases, down to the level of specific strains. The method is generalizable to isolate repositories across the infectious disease domain. Leveraging the other extension ontologies within the IDO suite, the method allows us to generate similar lattices for specific subfamilies of coronavirus-related diseases, influenza virus-related diseases, and so on. Together these form a larger network of infectious disease ontologies under IDO Core as illustrated in Fig. 5.

In this figure, where two ontologies are connected by an arrow, the one lower in the lattice extends, and imports needed terms from, the higher one, as well as from other ontologies higher up. To be clear, subontologies only import what is needed, not all of the terms and axioms from all the ontologies from which it draws. The ontologies at the very top are upper-level OBO ontologies from which IDO Core, and other ontologies further down in the lattice, extend. Note that the graph presents only a representative sample, rather than an exhaustive list, of upper-level ontologies upon which the lattice depends.

The remainder of our discussion focuses on a recent partitioning of the IDO suite of viral infectious disease ontologies under the *Virus Infectious Disease Ontology* (VIDO) and extensions covering coronavirus infectious diseases. We intend the work described below to serve as a model for the re-engineering of existing IDO Core extensions in such a way as to yield greater conformance with the ontology building principles discussed in the foregoing.

#### Virus Infectious Disease Ontology

VIDO [31] is a virus-neutral extension of IDO Core including terminological content used by researchers across various domains interested in the study of viral infectious diseases. VIDO thus provides a common language for IDO Core extensions covering viral infectious diseases such as IDOFLU and CIDO. Our example extensions from VIDO will focus on coronaviruses.

Like other IDO Core extensions, VIDO introduces terms from existing OBO Foundry ontologies where needed, such as OBI, NCBITaxon, and many others. From the NCBITaxon. VIDO imports the term *virus* and asserts it to be a subclass of *acellular structure*. VIDO also imports lower-level subclasses of *virus* from the NCBITaxon representing entities investigated by virologists such as *prion*, *viroid*, and *satellite*.

The NCBITaxon provides an exhaustive list of life science terms. However, three issues are worth noting when reusing NCBITaxon terms: First, with respect to virus terms NCBITaxon appears to align with the widely used International Committee on Taxonomy of Viruses (ICTV). However, ICTV guidance lacks systematic classification criteria and consequently leaves several viruses unclassified [49]. Second, when NCBITaxon is combined with automated importing tools such as the widely used Ontofox [50], this may result in the importing of an entire ICTV structured hierarchy – stretching from kingdom to species – resulting in large, unwieldy, taxonomies obscuring classes of interest. Third, NCBITaxon itself provides few textual definitions for terms. To align with OBO Foundry metadata conventions [51] and best practices [2], textual definitions and logical axioms are needed for *virus* and its subclasses.

These issues suggest that imported NCBITaxon terms should be supplemented with a more robust, simpler, ontological structure with accompanying textual definitions. The Baltimore Classification of viruses [52] – which groups viruses based on features of genetic structure – addresses both concerns, yielding seven, exhaustive, classes we import from the NCBITaxon as subclasses of *virus* corresponding to the Baltimore Classification.

**Fig. 6:**
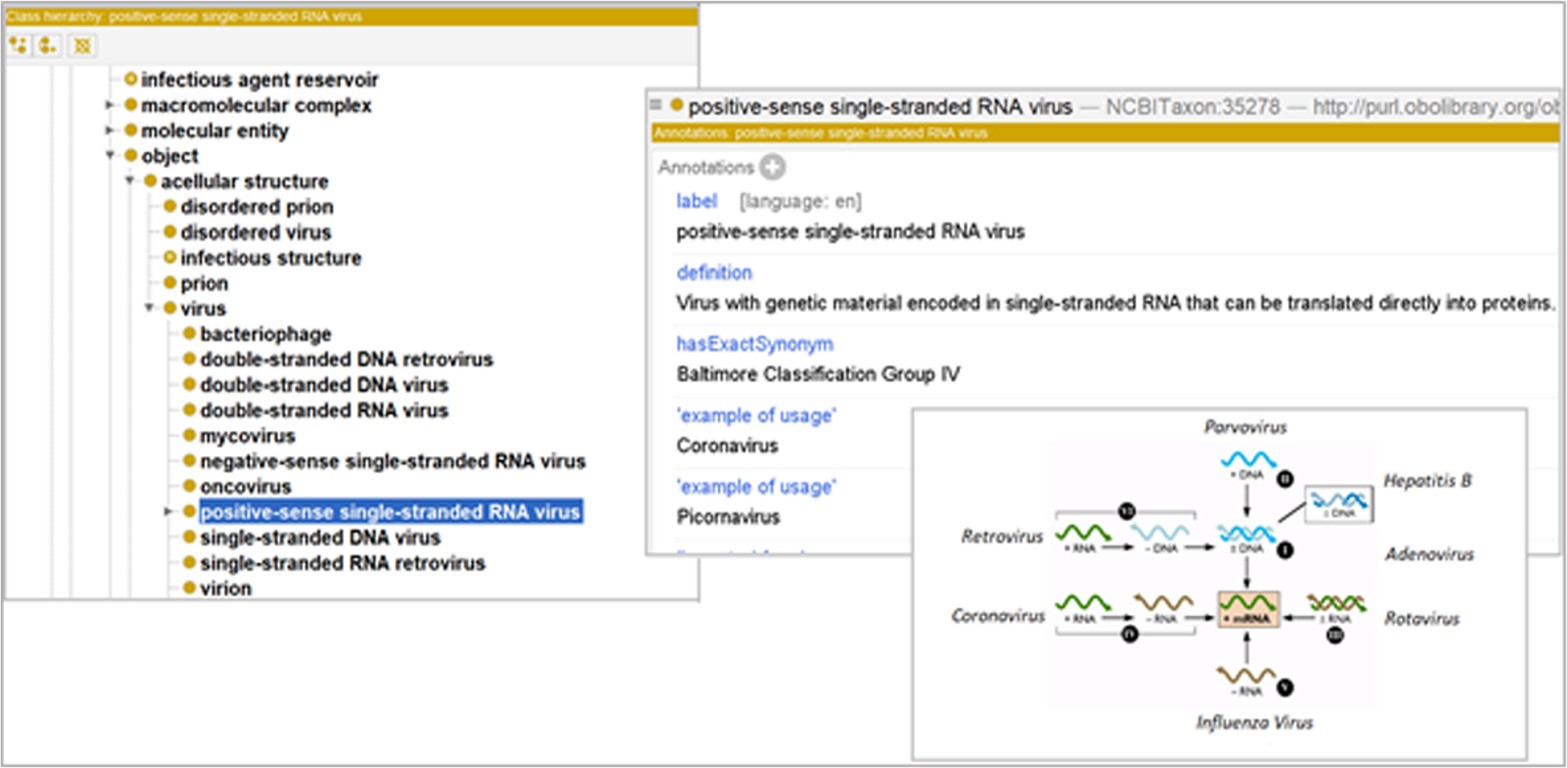
Protégé representation of Baltimore Classification

Figure 6 illustrates the Baltimore Classification in Protégé, supplemented by a standard visual summary of the seven viral replication pathways underwritten by virus genetic differences.

More generally, VIDO using the Baltimore Classification provides developers of more specific virus ontologies needed textual definitions, and a succinct, navigable, ontological structure which refers to viral replication pathways, and so to the obligate pathogenicity of viruses.

The IDO Core classes *infectious disorder*, *disease*, and *disease course* provide parent classes from which virus-specific children can be defined, as represented in Table 7 illustrating a simple recipe for extending IDO Core to a more specific domain.

**Table 7:** Virus and subclass definitions from VIDO

Term	Definition
*virus*	Acellular structure with RNA or DNA genetic material which uses host metabolic resources for RNA or DNA replication.
*positive-sense single-stranded RNA virus*	Virus with genetic material encoded in single-stranded RNA that can be translated directly into proteins.
*virus disorder*	Infectious disorder that exists as a result of a process of formation of a disorder initiated by a virus.
*viral disease*	Infectious disease inhering in a virus disorder that is a disorder due to the presence of the virus.
*viral disease course*	Infectious disease course whose physical basis is a virus disorder that is clinically abnormal in virtue of the presence of the relevant virus.
*symptomatic carrier role (IDO Core)*	Pathogen host role borne by an organism whose extended organism contains a pathogen bearing an infectious disposition towards the host, and the host has manifested symptoms of the infectious disease caused by the pathogen.
*asymptomatic carrier role (IDO Core)*	Pathogen host role borne by an organism whose extended organism contains a pathogen bearing an infectious disposition towards the host, and the host has no symptoms of the infectious disease caused by the pathogen.
*subclinical infection (IDO Core)*	Infection that is part of an asymptomatic carrier.
*subclinical virus infection*	Subclinical infection that is part of a virus host.

A given *virus disorder* is a material basis of some associated *viral disease* which may be realized in some associated *viral disease course*. Symptomatic cases of virus infection can be represented by importing terms from the Symptom Ontology, such as *dry cough*, *fever*, *taste alteration*, *smell alteration*, among others [53]. Worth noting is that these definitions are compatible with, for example, counting an asymptomatic carrier of SARS-CoV-2 as having the associated disease. This result aligns, moreover, with the CDC’s case criteria adopted on April 5th, 2020 which indicates that the presence of the SARS-CoV-2 genome or relevant antigens in an individual is sufficient to count as a case of COVID-19, and that asymptomatic cases should be counted as instances of the disease [54, 55].

Indeed, IDO Core already provides terms useful for distinguishing symptomatic and asymptomatic virus carriers, as well as subclinical infections from clinical infections, with relevant terms found in Table 7. The term *subclinical infection* reflects standard – if somewhat obscure – use of the terms “subclinical” and “asymptomatic” while nevertheless allowing for cases in which hosts with clinically abnormal infections exhibit no symptoms. For VIDO, this term is straightforwardly extended to *subclinical virus infection*, which is an infection caused by a virus that is part of an asymptomatic carrier.

#### The Coronavirus Infectious Disease Ontology

VIDO was developed as a bridge between IDO Core and extension ontologies representing specific diseases and specific causative pathogens. An extension of importance during the pandemic is the recently developed CIDO. Developed by Oliver He and his team, CIDO provides semantic resources needed for representing coronavirus genome, surveillance, vaccine, and host data. CIDO has been used to annotate 136 known anti-coronavirus drugs [56], identify 110 candidate drugs [22] for COVID-19 drug repurposing [57], and provides input to machine learning efforts [23] in identifying potential COVID-19 vaccines. Several members of both the IDO and VIDO development teams are also members of the CIDO development team working to ensure alignment among these ontologies, and adherence to OBO Foundry principles. Like VIDO, CIDO imports terms from a wide range of ontologies, including IDO Core, ChEBI [58], UBERON [59], GO, the Vaccine Ontology [60], and the NCBITaxon.

CIDO can straightforwardly extend from VIDO by adopting terms such as those in Table 8**.** More generally, CIDO can be populated by starting with a given virus term from VIDO, and then creating a subclass of that term restricted to members of the species coronavirus and associated diseases. Following representation of the Baltimore Classification in VIDO, for example, a subclass for *positive-sense single-stranded RNA virus* is a *coronavirus* which can be imported from the NCBITaxon, and for which a definition was generated above. Moreover, terms reflecting common features of coronaviruses can be imported from other OBO ontologies to characterize the virus species, such as that the viral genome including a five-prime nucleotide cap, or the common glycoprotein spikes found in the viral envelope [61, 62], many of which are represented in the Protein Ontology with terms such as *SARS-CoV-2 membrane protein* and *SARS-CoV-2 spike glycoprotein*.

**Table 8:** Extension of CIDO from VIDO

Term	Definition
*coronavirus*	Positive-sense single-stranded RNA virus with a helically symmetrical nucleocapsid, lipid bilayer viral envelope, and surface spike peplomers.
*coronavirus disorder*	Virus disorder that exists as a result of a process of formation of disorder initiated by a coronavirus.
*coronavirus disease*	Viral disease inhering in a coronavirus disorder.
*coronavirus disease course*	Viral disease course that is the realization of some coronavirus disease and has as a participant a coronavirus.

CIDO deals with coronavirus infectious diseases in general, and in that respect is more specific than VIDO. There are, however, several species of coronavirus which cause distinct infectious diseases, such as SARS-CoV-2 as the causative virus of COVID-19 and MERS-CoV as the causative virus of Middle-Eastern Respiratory Syndrome. Conformance with OBO guidelines requires ontologies be comprised of a small set of self-contained, reusable, terms and not unnecessarily duplicate terms found in other ontologies. There is a need in the present COVID-19 pandemic for terms specific to SARS-CoV-2 and COVID-19.

#### The COVID-19 Infectious Disease Ontology

**Fig. 7:**
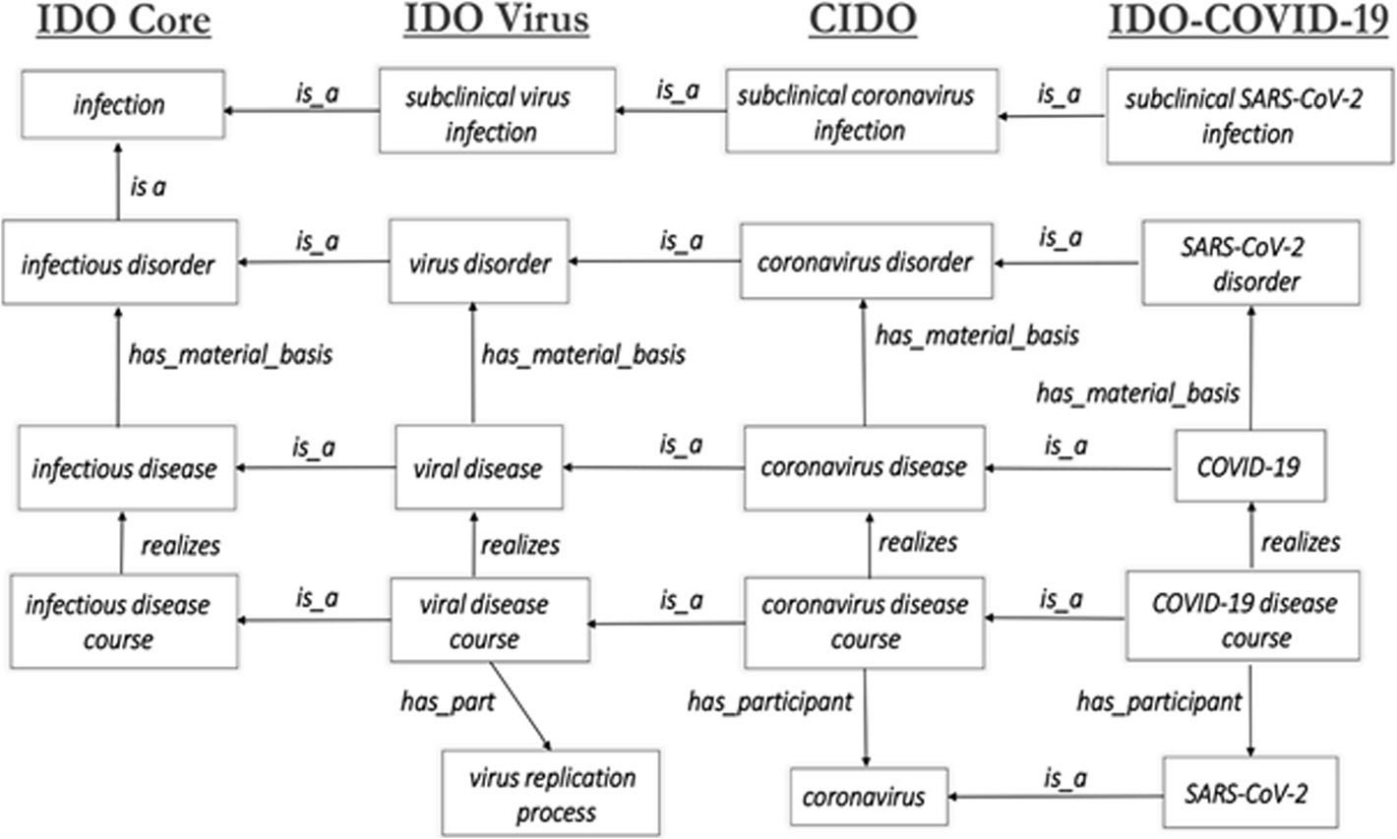
Links between VIDO, CIDO and IDO-COVID-19

IDO-COVID-19 extends from CIDO and covers COVID-19 and its cause SARS-CoV-2. IDO-COVID-19 thus brings together IDO Core, VIDO, and CIDO in the interest of fine-grained representation of this virus strain and associated diseases. Figure 7 summarizes links among these ontologies.

The starting point for IDO-COVID-19 is pathogenesis to COVID-19 caused by SARS-CoV-2. Flexibly representing COVID-19 pathogenesis is of importance during the current pandemic, as researchers are still working to understand how SARS-CoV-2 infections cause such a wide range of signs and symptoms across demographics. Representing COVID-19 pathogenesis in IDO-COVID-19 requires importing relevant terms from VIDO, CIDO, and relevant OBO Foundry ontologies, to define terms such as those found in Table 9. Instances of *SARS-CoV-2 pathogenesis* are in turn asserted as part of some *COVID-19 disease course*.

**Table 9:** Extension of IDO-COVID-19 from CIDO

Term	Definition
*COVID-19*	Coronavirus disease inhering in a SARS-CoV-2 disorder.
*COVID-19 disease course*	Coronavirus disease course that is the realization of some COVID-19 disease and has participant SARS-CoV-2.
*pathogenesis (GO)*	Process that generates the ability of a pathogen to induce a disorder in an organism.
*coronavirus pathogenesis (CIDO)*	Virus pathogenesis that is the realization of an infectious disposition inhering in a coronavirus or coronavirus population, having at least the process parts: (1) pathogen transmission, (2) establishment of localization in host, (3) process of establishing an infection, and (4) appearance of a virus disorder.
*SARS-CoV-2 pathogenesis*	Coronavirus pathogenesis process that is the realization of an infectious disposition inhering in SARS-CoV-2 or a SARS-CoV-2 population, having at least the proper process parts: (1) pathogen transmission, (2) establishment of localization in host, (3) process of establishing an infection, and (4) appearance of a virus disorder.
*replication (IDO Core)*	Production process in which a participant creates a copy of itself.
*virus replication (VIDO)*	Replication process in which a virus containing some portion of genetic material inherited from a parent virus is replicated.
*generative stage (IDO Core)*	Temporal subdivision of a developmental process.
*virus generative stage (VIDO)*	Infectious structure generative stage that is a temporal subdivision of a virus developmental process.
*virus attachment stage (VIDO)*	Virus generative stage during which a virion protein binds to molecules on the host surface or host cell surface projection.
*virus penetration stage (VIDO)*	Virus generative stage during which a virion or viral nucleic acid breaches the barriers of a host.
*SARS-CoV-2 attachment stage*	Virus attachment stage during which SARS-CoV-2 bonds with a host cell.
*SARS-CoV-2 penetration stage*	Virus penetration stage during which SARS-CoV-2 penetrates a host cell.
*SARS-CoV-2 adhesion susceptible cell*	Virus adhesion susceptible cell with a functional receptor part bearing an adhesion disposition realized in a SARS-CoV-2 attachment stage.
*SARS-CoV-2 penetration disposition*	Virus penetration disposition borne by a functional receptor complex that is the disposition to participate in a SARS-CoV-2 penetration process.
*negative regulation of SARS-CoV-2 attachment*	Negative regulation of coronavirus replication process that stops, prevents, or reduces the frequency of some SARS-CoV-2 attachment stage.
*negative regulation of SARS-CoV-2 penetration*	Negative regulation of coronavirus replication that stops, prevents, or reduces the frequency of some SARS-CoV-2 penetration stage.

The term *coronavirus pathogenesis* will ultimately be imported from CIDO, and is itself a subclass of the VIDO term *viral pathogenesis*, a subclass of *pathogenesis* imported from the Gene Ontology. As defined, 'pathogenesis' is a success term, in that it encompasses formation of disorder in an entity. This is reflected in (1)–(4) of the *SARS-CoV-2 pathogenesis* definition and motivated by the GO Consortium focus on canonical biological processes [4]. This is not to say all SARS-CoV-2 infections result in successful pathogenesis. An individual may be infected by SARS-CoV-2, but this need not result in a relevant disorder. Absent the relevant disorder, there is no appropriate material basis for COVID-19. Consequently, this would not count as an instance of *SARS-CoV-2 pathogenesis*, as the process part (4) would be missing.

Instances of *viral disease course* and *virus pathogenesis* have as respective parts *virus replication*. *SARS-CoV-2 pathogenesis* clearly involves replication in a host. The term *virus replication* is defined in VIDO as a subclass of the IDO Core term *replication*. IDO-COVID-19 imports the newly minted *generative stage* from IDO Core, defined as a temporal subdivision of a developmental process. Subclasses of which include the various stages through which viruses may proceed during a given replication.

Not all cells are susceptible to SARS-CoV-2 infection. In those cases of successful infection, the virus attaches to the alveolar epithelial cell with a spike surface glycoprotein, by way of these host cell’s angiotensin-converting enzyme 2 (ACE2) receptors [63, 64]. ACE2 receptors appear crucial for SARS-CoV-2 attachment, suggesting the need to define *SARS-CoV-2 adhesion susceptible cell*, which is a cell bearing an *adhesion disposition* realized in a *SARS-CoV-2 attachment stage*, where the functional receptor material base *ACE2* is imported to IDO-COVID-19 from the Protein Ontology [65] (from which it also imports recently created terms for SARS-CoV-2 proteins). A *SARS-CoV-2 attachment stage* is frequently followed by a penetration stage, involving penetration susceptible cells. More specifically, transmembrane protease serine 2 (TMPRSS2) aids in cleaving host cells in anticipation of SARS-CoV-2 fusing with the cell membrane [66], then introducing viral genomic RNA into the cytoplasm.

This similarly suggests a need to define *SARS-CoV-2 penetration susceptible cells* as cells bearing a *SARS-CoV-2 penetration disposition* where in this case the functional receptor material base is *TMPRSS2*, also imported to IDO-COVID-19 from the Protein Ontology. Reflection on other stages suggest corresponding terms, since following penetration, SARS-CoV-2 genome translation and virion assembly begins in the endoplasmic reticulum, forming virions then packaged into vesicles, sent to the host Golgi apparatus, and fused with the host cell membrane to exit the host. IDO-COVID-19 terms reflecting stages of the replication cycle for SARS-CoV-2 also provide targets for regulation of that cycle, important to vaccine, drug, and treatment options. Examples of negative regulation relevant here are *negative regulation of SARS-CoV-2 attachment* and *negative regulation of SARS-CoV-2 penetration*.

We should acknowledge that there are other ontology initiatives developed to support curation of COVID-19 data, such as the *WHO COVID-19 Rapid Version CRF* [67], the *COVID-19 Surveillance Ontology* [68], the *Linked COVID-19 Data Ontology* [69], and the NASA Jet Propulsion Laboratory’s *COVID-19 Research Knowledge Graph* [70]. However, since each is a stand-alone initiative developed outside the scope of OBO Foundry principles, each is subject to the silo problems documented in the introduction.

## Discussion

Since IDO Core is built in accordance with the OBO Foundry principles, this means that the IDO ontologies are interoperable with other OBO Foundry ontologies. IDO Core, VIDO, CIDO, and IDO-COVID-19, for example employ a well-specified syntax, common identifiers, and a common top-level ontology, as required by the Foundry. Each is openly available in the public domain under creative commons licenses as well. Recognizing that these ontologies are in neighboring domains, developers have worked closely to ensure each ontology is modular and remains within its clearly specified scope using unambiguous terms. Collaboration has taken the form of publications [31], conference presentations, and weekly harmonization meetings.

**Fig. 8:**
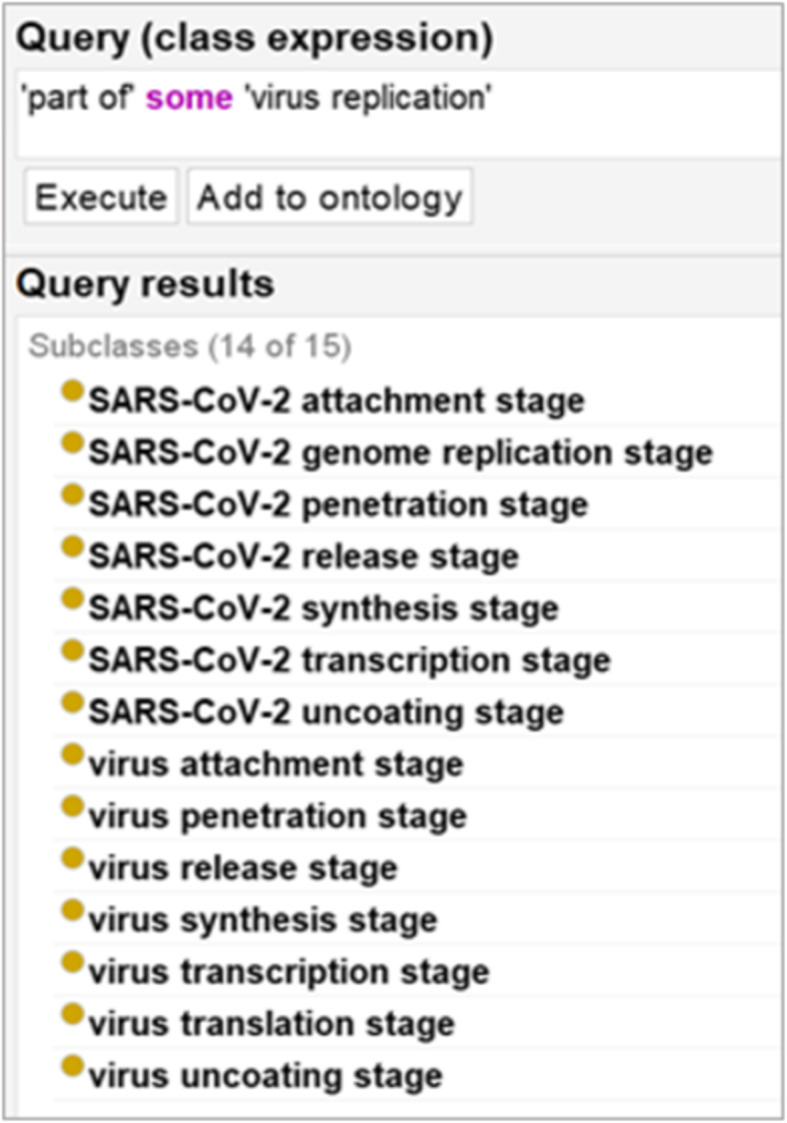
DL Query for *part_of* some *virus replication*

**Fig. 9:**
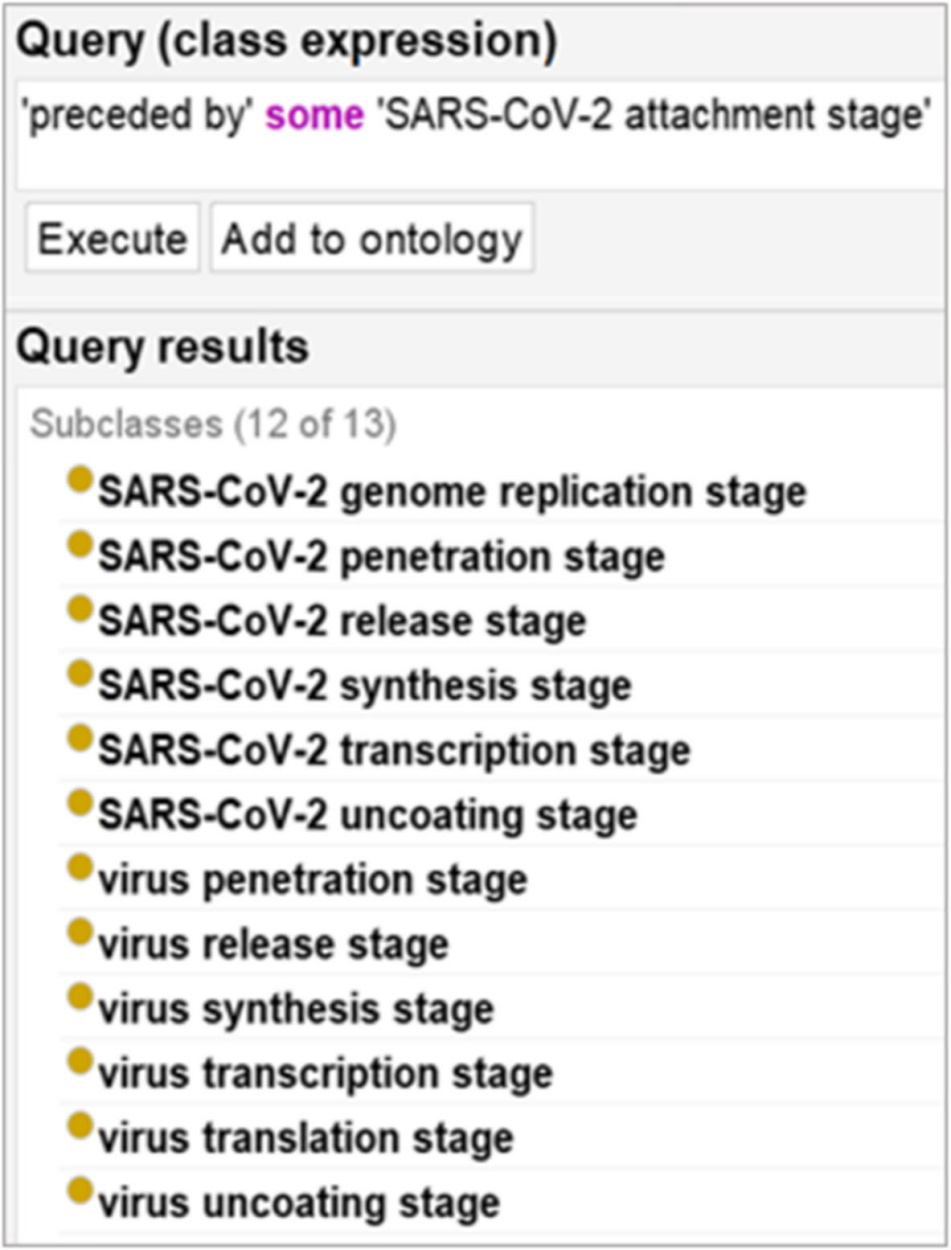
DL Query for *preceded*_*by* some *SARS-CoV-2 attachment stage*

Ontology metadata can be used to combine heterogeneous bodies of research data to enable structured querying and analysis [71]. Figure 8 and Fig. 9, illustrate, for example, simple Description Logic queries of IDO-COVID-19. The former returning any classes instances of which are occurrent parts of virus replication processes, while the latter returns any class instances of which are preceded by some SARS-CoV-2 attachment stage. As has been revealed by the COVID-19 pandemic, failure to pay heed to metadata standards limits the reusability of available primary genomic data, significantly impeding efficient response measures [72].

Adherence to Foundry principles makes the IDO ontologies applicable to the annotation of a variety of databases relevant to infectious disease that already make use of Foundry ontologies in their annotations [18]. For examples of databases to which IDO ontology annotations have been previously applied, see Supplementary Table 5.

VIDO, CIDO and IDO-COVID-19 are currently being used to annotate approximately 400 articles in the National Library of Medicine [73] COVID corpus, which report COVID-19 clinical trial, epidemiological, and pathogenesis data. The resulting ‘gold standard’ corpus will be used to train algorithms for automated annotating tasks. These algorithms will in turn be used to identify useful patterns in COVID-19 datasets on the model illustrated in [74], which describes a novel method for learning features of entities such as proteins and viruses from their associations to ontology classes, and describes how this method can be employed for fast identification of virus–host interactions that can shed light on potential treatments and drug discoveries. That said, this work is in its infancy and we hope to report our results in future work.

In the ideal case data and information relevant to infectious disease research, independently of where they are stored, should be annotated using IDO terms. The resultant annotated data would thereby become available to computer processing as if they formed a single body of linked data in virtue of the semantically controlled properties of the IDO terms and of the logical structure of their definitions. Experience shows, however, that these benefits are difficult to achieve except in those cases where databases have been created using the ontology structure from the very beginning, an approach pursued most successfully in the case of the incorporation of Gene Ontology annotations into the UniProt database [75, 76] provides an illustration of this approach in the field of influenza research. Matters are improving in this respect with the development of approaches to data annotation using machine learning. The results are still in many cases disappointing, but they are at least improving over time [77].

To accelerate these improvements, it will be necessary to associate with each OBO Foundry ontology a terminology comprising, for example, (1) those terms in common usage in the relevant literature that denote entities which are denoted by different terms in the ontology, (2) terms denoting entities that are more specific than are covered in the salient ontology. This will then require a special set of relationships to indicate, for any given term in the terminology, the nature of the annotation with an ontology term. Where ontology precedes data, annotation then becomes automatic.

All too often, however, problems arise, for example, because it is too difficult to associate terms from the controlled vocabulary with the terms used by those responsible for data collection. Terms in databases and literature may denote instances or types by using the exact same term that is used in an ontology to denote a perhaps related, but still different type. Furthermore, databases and literature may use terms that denote entities which in the ontology are denoted by different terms. Even more prevalent are terms in databases that denote entities more specific than those covered in an ontology. This requires a special set of relationships to indicate the nature of the annotation with the ontology term. In future work we hope to explore the extent to which the ontology structure of the IDO suite can enhance the construction of infectious disease databases by using the “ontology precedes data” approach employed with success by the Gene Ontology.

Admittedly, not all IDO extension ontologies have adequately adhered to the IDO strategy as presented in the foregoing. Part of the goal of our current work on CIDO, VIDO, and IDO-COVID-19 is to provide a model according to which other IDO extension ontologies can be brought into tighter coordination with the Core, as well as an easy-to-follow recipe for building new pathogen- specific ontologies so that infectious disease researchers are given fewer opportunities to generate inoperable ontologies. As we continue to face the threat of novel viruses (as well as bacteria and parasites) in the future, having such a blueprint in hand should facilitate more rapid extension of the IDO suite.

Relatedly, ontology annotations are all too often applied incorrectly. Many users of OBO Foundry ontologies do not seem to understand BFO, OGMS, or the principles upon which they are based. This suggests the OBO community needs to work harder to make sure these principles are well understood. And even where the principles are more or less understood, it is likely we need to be more vigilant in ensuring OBO Foundry users are actually complying with them. A complete solution to these issues is beyond the scope of this paper, though we intend our work here to illustrate some guidance to users working with OBO ontologies. Users should develop a firm understanding of the classes, relations, and principles of BFO, which can be fostered both by studying existing user guides [2, 78] and online tutorials [79], and by signing up for and participating in the BFO user group [80]. Users should, moreover, develop competence in OBO methodological principles by reviewing the extensive guidance from the OBO Foundry website [6], Github issue tracker [81], and perhaps signing up for the OBO Slack channel [82] for discussion. Users should, additionally, understand the goals of ontology use, and how proper integration of a small, narrowly focused ontology, with other small ontologies, can result in significant semantic resources for existing domains and those yet to be represented. Internalizing the preceding information will provide a crucial foundation for the proper use of extensions of BFO in the OBO Foundry. Of course, users competent with BFO and OBO principles must be able to rely on other ontologists working with and building OBO ontologies. Ontologies that are counted in the OBO library which are not designed in conformance with OBO principles undermine OBO standards, and may lead to confusion among users. With that in mind, the OBO operations committee members and working group should play a more active role in ensuring OBO ontologies align with OBO principles. There should, perhaps, be stakes for allowing one’s ontology to fall out of conformance with these standards, e.g. loss of membership in the library. Members of the OBO committees might schedule, for instance, routine inspections of ontologies to determine whether they align with OBO standards. From another direction, the OBO library might be given conformance or reliability ratings by committees, much like restaurants are given scores and organizations are given credit ratings. OBO already institutes something like this practice. There is presently the OBO Foundry - a small group of ontologies vetted by the more active members in the OBO community - and the broader OBO library - ontologies that met initial OBO standards for inclusion. Further tiers might be constructed so that users are more easily able to identify the paradigms of good ontology development, and consequently, use those ontologies to guide their own ontology development.

The lattice network, illustrated above, can be used to define a strategy for constructing a taxonomy of infectious diseases incorporating both high- throughput genetic and molecular data as well as clinical data. The network can also be used for rapidly creating new ontologies for novel pathogens or novel strains in a way that provides a pathway for automatic linking of emerging data to legacy data relating to existing pathogens and diseases. The IDO suite of ontologies can thereby contribute to the advance of what is called ‘personalized’ or ‘precision’ medicine, which depends upon effective classification and association of biological disease data with known clinical phenotypes and disease types at ever finer levels of detail.

One might worry our lattice methodology may lead to a combinatorial explosion of ontologies. For example, the lattice of *S. aureus* infectious disease ontologies [42] suggests distinct ontologies will be needed for each strain, host, and so forth. In response, note application ontologies are added to the lattice if there is a need from researchers describing genuine biological phenomena, not simply because of combinatorial possibilities. Even so, one may worry that our lattice methodology coupled with advances in personalized medicine may lead to an explosion of personalized ontologies.

More specifically, representing individual patients may require fine-grained ontologies, with substantial overlap, and minor differences. In response, we find this a feature rather than a bug of our methodology. First, and again, if there is a need for personalized ontologies then we intend to be compatible with that need. Second, though personalized ontologies would perhaps overlap substantially, they will also be substantially distinct. For example, Sally and John both bear temperature as a determinable, but each bears a distinct temperature as a determinate quality. Similarly for mass, pathogen immunity, respiratory capacity, and so forth. Moreover, the token individual Sally will be distinct from the token individual John.

These remarks apply equally to newly emerging pathogens. For example, suppose we need a SARS-CoV-3-focused ontology. We then import from IDO Core, OBI, VIDO, CIDO and other ontologies, define what terminological content we can from imported terms. We introduce the virus SARS-CoV-3 as a primitive subclass of coronavirus. The result is an ontology largely composed of existing ontologies, with a proper part composed of combinations of that new primitive with existing terms—for example, SARS-CoV-3 infection, SARS-CoV-3 disorder, and so on. And, since by assumption, we need to represent SARS-CoV-3 data, we are justified in adding them. We should be as specific as researchers need.

Implementation of the lattice methodology requires significant maintenance and overhead to keep IDO Core, the mid-level ontologies, and the various sub-ontologies all in sync. If a definition or relationship changes in IDO Core, developers of extending ontologies will need to be notified. And if IDO Core changes in such a way that some of the axioms in downstream ontologies might become inconsistent, there should be some process by which this can be detected and resolved. Aware of such issues, we have created a Github Organization, where developers of IDO extension ontologies can discuss needs in the IDO ecosystem, coordinate together on updates, and are alerted to changes in ontologies populating the IDO organization [83]. The organization follows maintenance protocols modeled on OBO Foundry principles. While we do not at present implement any software tools to support automatic updates, we hope to explore the development of such tools in future work.

## Conclusions

As we face the continued threat of novel pathogens in the future, IDO Core provides a simple recipe for building new pathogen-specific ontologies in a way that allows data about novel diseases to be easily compared, along multiple dimensions, with data represented by existing disease ontologies. The IDO strategy, moreover, supports ontology coordination, providing a powerful method of data integration and sharing that will allow physicians, researchers, and public health organizations to respond rapidly and efficiently both to the current and future public health crises.

## Methods

With respect to editing tool, IDO Core was updated using the Protégé ontology development tool [84], leveraging the enhanced expressivity of the Web Ontology Language (OWL). Ontologies were tested against automated reasoners such as HermiT and Pellet. Additionally, logical axioms underwriting these ontologies were translated into a syntax readable by the Mace4 model checker, which allowed for manual graphical inspection of classes of models constrained by the asserted axioms. An automated proof-checker Prover9 bundled with Mace4 was used to validate expected theorems while refining axiom models.

IDO Core, like other OBO Foundry ontologies, is not exhaustive, as development of the ontology is intended to maintain pace with growing research on infectious diseases. With respect to updating IDO Core based on the existing OBO library, a study of extension ontologies was conducted in the interest of identifying terms in extensions that would be better placed in IDO Core. From another direction, a study of developments in OBO Foundry ontologies was conducted in the interest of identifying terms better suited to more general ontologies. In the event terms were needed for IDO Core which were not suitable for introduction, because too general for the domain of infectious diseases, term requests were made to developers of relevant OBO Foundry ontologies. For example, transmission classes were requested for and subsequently added to the Pathogen Transmission Ontology. Lastly, with respect to updating IDO Core based on the construction of novel reference ontologies, such as VIDO, collaborative study between IDO Core, VIDO, CIDO, and IDO-COVID-19 developers resulted in careful construction of relevant terms based on up-to-date empirical literature, researcher term use, and logical coherence. For example, adjustments were needed to IDO Core’s definition of *infectious agent* due to reflection on viruses, resulting in the introduction of the class *acellular structure* as parent class to *virus*.

In every case, terminological content for IDO Core was either imported from an existing OBO ontology, defined based on imported terms, introduced as a primitive to IDO Core, or defined based on IDO Core primitive and/or imported terms. In accordance with OBO Foundry principles, priority was given to importing and defining terms, over introducing primitive terms to IDO Core. Before new primitives were deemed necessary, IDO Core developers canvased researchers developing nearby ontologies for insights, posed queries on issue trackers on relevant GitHub pages, and studied relevant infectious disease literature. Terms were then introduced, vetted by specialists where possible, then introduced to IDO Core after scrutiny.

As with most OBO ontologies, IDO Core is an open project with its own GitHub repository [85], where the most recent published and developmental versions of the ontology are available for download. We encourage members of the ontology community, as well as infectious disease researchers, to submit term requests to our GitHub Issues tracker. The Issues tracker can also be used to report any errors or concerns related to the ontology. Before requesting a new term, please search online ontology repositories such as Ontobee and BioPortal to see if the needed term already exists. Once a term request is received, it will be reviewed by the main IDO Core team to determine whether the term is most appropriate for IDO Core, one of its extensions, or another OBO ontology. If the term is within IDO Core’s scope, then it will be added with a formal definition, written in conjunction with the term requestor to ensure biological accuracy as well as adherence to OBO Foundry best practices and consistency with IDO logical structure. We can assign a unique ID for the term so that it can be used for immediate annotation prior to the definition being finalized.

## Supplementary Information

**Additional file 1. **Supplementary Tables and Related Discussion. **Table S1.** Ontologies building on the OGMS treatment of disease and diagnosis; **Table S2.** Overview of IDO extension ontologies that have been developed or planned; **Table S3.** Some other ontologies within the infectious disease domain that make use of IDO Core. **Table S4.** IDOBRU Hierarchy; **Table S5.** Some databases to which IDO annotations have been applied; **Table S6.** IDO based Decision Support Systems

**Additional file 2.** The Infectious Disease Ontology Extensions: Some Issues. (.docx). Several IDO ontologies require significant reengineering if they are to be considered bona fide extensions of IDO Core. This document provides an overview of some issues concerning specific IDO extensions, while providing some suggestions for how they can be addressed.

**Additional file 3: Case Study.** IDOSA and methicillin resistant *Staphylococcus aureus* (.docx)

## Data Availability

The datasets generated and/or analysed during the current study are freely publicly available in the IDO Core GitHub repository [https://github.com/infectious-disease-ontology/infectious-disease-ontology] as well as online ontology repositories such as Ontobee. [http://www.ontobee.org/ontology/IDO] and BioPortal [http://www.ontobee.org/ontology/IDO]. IDO extensions are also freely publicly available on Github, Ontobee and BioPortal.

## Abbreviations

Apollo-SV: Apollo Structured Vocabulary
BFO: Basic Formal Ontology
CIDO: Coronavirus Infectious Disease Ontology
ChEBI: Chemical Entities of Biological Interest
CL: Cell Ontology
GO: Gene Ontology
IDOBRU: Brucellosis Ontology
IDO Core: Infectious Disease Ontology Core
IDODEN: Dengue Fever Ontology
IDOFLU: Influenza Ontology
IDOHIV: HIV Ontology
IDOMAL: Malaria Ontology
IDOMEN: Meningitis Ontology
IDOPlant: Plant Disease Ontology
IDOSCHISTO: Schistosomiasis Ontology
IDOSA: *Staphylococcus aureus* Infectious Disease Ontology
IAO: Information Artifact Ontology
NARSA: Network on Antimicrobial Resistance in *Staphylococcus aureus*
NCBITaxon: NCBI organismal classification
OBI: Ontology for Biomedical Investigations
OBO: Open Biomedical Ontologies
OGMS: Ontology for General Medical Science
OWL: Web Ontology Language
RO: Relations Ontology
VIDO: IDO Virus

## Footnotes

1 Individuals with CCR-5 mutations do exhibit *other* clinical abnormalities, and so disorders, but importantly, this is not due to the HIV-1 virus. Rather, it is due to the genetic mutation.

2 For example, commensal oral bacteria may infect the bloodstream following damage caused by vigorous brushing. Yeast overgrowth may occur when a healthy balance of defensive bacteria is reduced in the host.

3 For IDO Core we have modified the textual definitions from their originals to align with BFO principles [2].
