# Supplementary material for: The Infectious Disease Ontology in the age of COVID-19
Source: J Biomed Semantics. 2021 Jul 18;12:13. doi: 10.1186/s13326-021-00245-1 (PMC8286442; doi:10.1186/s13326-021-00245-1)
Supplement: Supplementary file 1 — Additional file 1. Supplementary Tables and Related Discussion. Table S1. Ontologies building on the OGMS treatment of disease and diagnosis; Table S2. Overview of IDO extension ontologies that have been developed or planned; Table S3. Some other ontologies within the infectious disease domain that make use of IDO Core. Table S4. IDOBRU Hierarchy; Table S5. Some databases to which IDO annotations have been applied; Table S6. IDO based Decision Support Systems [file 13326_2021_245_MOESM1_ESM.docx]

The Ontology for General Medical Science (OGMS) covers types of entities relevant to clinical encounters between doctor and patient. Thus, it includes representations of disease, causes and manifestations of disease, diagnosis, symptom, treatment, patient examination, history taking, laboratory test, and so forth.

**Table S1** **|** Ontologies building on the OGMS treatment of disease and diagnosis

| Agronomy Ontology (Agro) [1] | Apollo Structured Vocabulary (Apollo-SV) [2] |
| --- | --- |
| Beta Cell Genomics Ontology (BCGO) [3] | Cardiovascular Disease Ontology (CVDO) [4] |
| Coronavirus Infectious Disease Ontology (CIDO) [5] | Human Disease Ontology (DOID) [6] |
| Drug Ontology (DrugON) [7] | VEuPathDB ontology (EUPATH) [8] |
| Influenza Ontology (IDOFLU) [9] | Genomic Epidemiology Ontology (GenEpio) [10] |
| Hypertension Ontology (HTN) [11] | Infectious Disease Ontology (IDO) [12] |
| Brucellosis Ontology (IDOBRU) [13] | Meningitis Ontology (IDOMEN) [14] |
| Plant Disease Ontology (IDOPlant) [15] | *Staph aureus* Infectious Disease Ontology (IDOSA) [16] |
| Schistosomiasis Ontology (IDOSCHISTO) [17] | Mental Functioning Ontology (MFO) [18] |
| Mental Diseases Ontology (MFOMD) [19] | Monarch Disease Ontology (MonDO) [20] |
| Neurological Disease Ontology (ND) [21] | Ontology of Adverse Events (OAE) [22] |
| Ontology for Biomedical Investigations (OBI) [23] | OBI-NIAID-GSC-BRC-view [24] |
| Ontology for Biobanking (OBIB) [25] | Ocular Disease Ontology (ODO) [26] |
| Oral Health and Disease Ontology (OHD) [27] | Ontology of Host Pathogen Interactions (OHPI) [28] |
| Ontology of Laboratory Animal Medicine (OLAM) [29] | Ontologized MIABIS (OMIABIS) [30] |
| Ontology of Medically Relevant Social Entities (OMRSE) [31] | Obstetric and Neonatal Ontology (ONTONEO) [32] |
| Ontology of Precision Medicine and Investigation (OPMI) [33] | Prescription of Drugs Ontology (PDRO) [34] |
| Planarian Phenotype Ontology (PLANP) [35] | Vaccine Ontology (VO) [36] |
| Vital Sign Ontology (VSO) [37] |  |

**Table S2** **|** Overview of IDO extension ontologies that have been developed or planned

| Malaria Ontology (IDOMAL) [38] | Created by the Christos Louis team at the Institute of Molecular Biology and Biochemistry (IMBB) in Crete, Greece, IDOMAL covers both clinical and epidemiological aspects of malaria, disease and vector biology, as well as intervention attempts to control the disease. IDOMAL was originally developed in the context of VectorBase. |
| --- | --- |
| Dengue Ontology (IDODEN) [39] | Also developed by the Louis research team at IMBB, IDODEN covers all aspects of dengue fever including disease biology, epidemiology, clinical features, and vector entomology. IDODEN was developed in the context of VectorBase and its structure was designed to mirror that of IDOMAL. Intended for use in Dengue decision support systems. |
| Brucellosis Ontology (IDOBRU) [13] | Developed by Yongqun He and his team at the University of Michigan, IDOBRU focuses on Brucellosis, a highly contagious zoonotic infectious disease caused by the intracellular, Gram-negative bacteria *Brucella*. IDOBRU encompasses the domains of clinical care, public health, and biomedical research, along seven major axes: host infection and zoonotic disease transmission, symptoms, virulence factors and pathogenesis, diagnosis, intentional release, vaccine prevention, and treatment. |
| Influenza Ontology  (IDOFLU) [9] | Developed by Richard Scheuermann, Lynn Schriml, Joane Luciano, and Burke Squires, IDOFLU covers the natural, experimental and clinical realms related to influenza virus life cycle, infection and disease. IDOFLU utilizes OBI classes for components of materials, qualities, and processes to map influenza virus sequence and surveillance terms to their corresponding materials and qualities. IDOFLU is applied to data collected by the Centers for Excellence in Influenza Research and Surveillance (CEIRS) project to help researchers more easily elucidate influenza virulence and pathogenesis etiology. |
| *Staphylococcus aureus* Infectious Disease Ontology (IDOSA) [16] | Developed by Lindsay Cowell, Barry Smith and Albert Goldfain, in collaboration with Dr. Vance Fowler at Duke University Medical Center, IDOSA focuses on Staph aureus (Sa) infection diseases. The ontology is used to analyze networks of functionally related gene products to identify host genes conferring susceptibility to Staphylococcus aureus bacteremia and has the potential to provide a classification of Sa that allows automated inference of resistance profiles. |
| HIV Ontology (IDOHIV) | Developed by Martin Schiller of UNLV, the HIV ontology is intended to cover all types of HIV data and information; intended for use in the HIVToolbox web application [40]. |
| Schistosomiasis Ontology (IDOSCHISTO) [17] | Developed by team of researchers based in Senegal led by Dr. Gaoussou Camara, IDOSCHISTO focuses on schistosomiasis, a waterborne infectious disease caused by *Schistosoma* helminth parasites. The ontology is organized into 3 main sub-modules; i) Biology, e.g. pathogen/host interactions, host physiological reactions to the disease, pathogen taxonomy and life-cycle; ii) Epidemiology, for example risk factors, spread of disease, means to prevent and control; and iii) Clinical, including symptoms that influence differential diagnoses and treatment decisions. |
| Plant Disease Ontology (IDOPlant) [15] | Under active development within the context of the Planteome Project, IDOPlant provides a comprehensive reference ontology for any infectious plant disease. The main aims of IDOPlant are to “provide plant scientists with the means identify genomic and genetic signatures host-pathogen interactions, resistance, or susceptibility, and to help agronomists and farmers by developing tools to identify disease phenotypes and gather epidemiological statistics” [41]. |
| Meningitis Ontology (IDOMEN) [14] | Also developed by Dr. Camara’s team, IDOMEN focuses on meningitis, a disease caused by the gram-negative bacteria, *Neisseria meningitidis*. IDOMEN covers the meningitis domain along three main axes: i) biological (immunity, virulence factors, pathogen and host biology); ii) clinical (clinical manifestations, laboratory tests and findings, diagnosis and treatment); iii) epidemiological (surveillance, prevention, epidemic emergence factors such as risk behaviors, climate and environment). IDOMEN is designed to assist in the analysis and filtering of data collected on social media platforms such as Twitter to help improve the early detection of meningitis epidemic risks in sub-Saharan Africa. Increasingly, search queries, social media posts, and web server access logs have been employed for disease surveillance. Digital platforms provide real time data streams from which information related to public health can be extracted in a timely manner at low to virtually no cost. By mining these data sources for traces of health-related activities, they can be transformed into useful metrics for inclusion in statistical estimation models for disease incidence. Google, Wikipedia and Twitter have each been investigated as tools for quantifying disease incidence rates [42-44]. Ontology-based approaches to internet-based disease surveillance have also been developed and applied with good success [45, 46]. |
| Coronavirus  Infectious Disease  Ontology (CIDO) [5] | Initiated by Yongqun He and Hong Yu, CIDO was developed by a collaborative group of researchers in both the US and in China in response to the recent COVID-19 outbreaks. CIDO provides a standardized human- and computer-interpretable annotation and representation of various coronavirus infectious diseases including their etiology, transmission, epidemiology, pathogenesis, diagnosis, prevention and treatment. |
| IDO Tuberculosis (IDOTUB) | Planned, but is not yet currently in development. |
| IDO Infective Endocarditis | Planned, but not yet currently in development. Preliminary work is available online: https://sivsmusings. wordpress.com/ |

**Table S3** **|** Some other ontologies within the infectious disease domain that make use of IDO Core

| Vaccine Ontology (VO) [36] | Developed in collaboration with the IDO Core team, VO has strong ties to the IDO initiative. Maintained by the He team at the University of Michigan, VO focuses on the classification of vaccines and vaccine components, vaccine quality and phenotypes, and host immune response to vaccines. VO is used in the Vaccine Investigation and Online Information Network (VIOLIN, http://www.violinet.org/) a central repository for literature related to, and data resulting from, vaccine research. |
| --- | --- |
| Bacterial Clinical  Infectious Disease  Ontology (BCIDO) [47] | BCIDO provides a controlled terminology for clinical bacterial infectious diseases along with domain knowledge commonly used in the hospital in-patient setting. BCIDO was designed to augment the use of ADSSs, thus serving as a tool to guide differential bacterial diagnoses and to assist in the prescribing of appropriate antimicrobial treatments. As such, BCIDO “encompasses terms and knowledge about common clinical presentations of [bacterial] infections, patient specific factors that influence differential diagnoses and treatment options, the [bacteria] themselves, and the antimicrobial agents used to treat infections” [47]. Though not an IDO extension per se, BCIDO imports IDO Core in full. |
| Vector Surveillance and Management Ontology (VSMO) [48] | Developed by a team of researchers at Colorado State University led by Drs. Lars Eisen and Saul Lozano, in collaboration with Dr. Cowell, VSMO covers the domain of surveillance and management of vectors and vector-borne pathogens, with special emphasis on content to support operational activities through inclusion in databases, data management systems and decision support systems [48]. The ontology includes terms for i) arthropod species capable of being biological vectors and for pathogen species transmitted by arthropod vectors; ii) chemical compounds relevant to insecticide resistance and chemical pesticide active substances (originating from MIRO); iii) terms for equipment used to collect or control vectors or vertebrae pathogen hosts, and tools used to kill vectors or prevent contact with humans. In consultation with the IDO Core developers, the relation *has_vector* was created for VSMO to link pathogens to their biological vectors. |
| Genomic Epidemiology Ontology  (GenEpio) [10] | GenEpio is a controlled vocabulary for infectious disease surveillance and outbreak investigations implementing whole genome sequencing (WGS) of microbial pathogens. GenEpio aims to enable the integration and promotion of all contextual information required to interpret pathogen genomics data, including critical knowledge about sequencing pipelines and sequence quality; lab results describing antimicrobial resistance and virulence phenotypes; epidemiological data concerning potential sources of risk and exposure; as well as data about susceptible populations and geographical distributions of pathogen strain [10]. GenEpio is currently being integrated into IRIDA (Integrated Rapid Infectious Disease Analysis), a user-friendly, decentralized, open-source bioinformatics and analytical web platform to support real-time infectious disease outbreak investigations using WGS data [49]. |

**Examples: IDOBRU & IDOPlant**

Two ontologies from **Table S2** provide examples of excellent ontology design. IDOBRU, the Brucellosis Infectious Disease Ontology, is maintained by the He research team at the University of Michigan, and is used to facilitate the integration and exchange of brucellosis information stored in widely used databases, including:

The *Brucella* Bioinformatics Portal [50]: a portal for the search and analysis of individual *Brucella* genes; linked to more than 20 other databases and programs.

The Vaccine Investigation and Online Information Network (VIOLIN) [51] a central repository for literature related to, and data resulting from, vaccine research.

IDOBRU exhibits a well-organized hierarchy with BFO, OGMS, and IDO Core imported in full, and is a good exemplar of the IDO Core hub and spokes model, as illustrated in **Table S4**.

**Table S4 | IDOBRU Hierarchy**

| IDOBRU Axis | Top Level IDOBRU Classes | Imported OBO Ontology Class from which it descends |
| --- | --- | --- |
| host infection and zoonotic disease transmission | *process of establishing* *Brucella infection in host*,  *Brucella infectious disposition*,  *Brucella* *host role* | *process of establishing an infection* (IDO Core)  *zoonotic disposition* (IDO Core)  *infectious agent host role* (IDO Core) |
| virulence factors and pathogenesis | *Brucella virulence factor*,  *Brucella virulence factor disposition* | *virulence factor* (IDO Core),  *virulence factor disposition* (IDO Core), |
| symptoms | *brucellosis symptom* | *symptom* (OGMS) |
| diagnosis | *brucellosis diagnosis* | *diagnosis* (OGMS) |
| intentional release | *Brucella intentional release* | *planned process* (OBI) |
| vaccine prevention | *brucellosis vaccine* | *vaccine* (VO) |
| treatment | *brucellosis treatment* | *treatment* (OGMS) |

The other example worth noting is IDOPlant, [15] a plant infectious disease ontology being developed under the auspices of the Planteome Project, which maintains a large database of annotations from plant genomic and phenomic studies [52]. IDOPlant leverages IDO Core in axioms such as the following:

IDOPlant:*process of establishing a Xanthomonas oryzae infection* subclass-of IDOPlant*:process of establishing a plant bacterial infection*,

IDOPlant*:process of establishing a plant bacterial infection* subclass-of IDO*:process of establishing an infection*

IDOPlant:*rice bacterial leaf blight disease* subclass-of IDOPlant:*plant bacterial disease*

IDOPlant:*plant bacterial disease* subclass-of IDOPlant:*plant infectious disease*

IDOPlant:*plant infectious disease* subclass-of IDO:*infectious disease*

**Table S5 | Some databases to which IDO annotations have been applied**

| The Eukaryotic Pathogen Genomics Database (EuPathDB) [53] | Provides genomic and other data for eukaryotic pathogens including *Cryptosporidium*, *Giardia*, *Plasmodium*, *Theileria*, *Toxoplasma*, and *Trichomonas* strains. Maintained by a team of researchers at the University of Pennsylvania led by Chris Stoeckert. |
| --- | --- |
| VectorBase [54] | Provides genomic and other data for a variety of invertabrae vectors of human pathogens. Also maintained by the Stoeckert team. |
| Eukaryotic Pathogen, Host & Vector Genomics Resource (VeuPathDB) | With support from a recently awarded 5-year contract with the National Institute of Allergy and Infectious Diseases, worth up to $7.2 million in 2019-2020 [55], the Stoeckert team has integrated EuPathDB and VectorBase into one bioinformatics resource, VeuPathDB. IDO Core is playing a role in this project, as the VeuPathDB application ontology [56] imports several IDO Core terms such as: *human pathogenicity disposition*, *infection*, *infection prevalence*, and *primary infection*, each of which are used in the annotation of VeuPathDB datasets. |
| Influenza Research Database [57] | Resource to elucidate host-influenza virus interactions, leading to new treatments and preventive action. Contains “surveillance data, human clinical data associated with virus extracts, phenotypic characteristics of viruses isolated from extracts, and all genomic and proteomic data available in public repositories for influenza viruses” [58]. |
| Virus Pathogen Resource [59] | Database and analysis resource for human pathogenic viruses, including sequence, surveillance and host response data. |
| PHIDIAS [60] | PHIDIAS (Pathogen-Host Interaction Data Integration and Analysis System) is a web-based database system for searching, comparing, and analyzing integrated genome sequences, conserved domains, and gene expression data related to pathogen-host interactions [60]. |
| Victors virulence factors database [60] | A database to store and analyze virulence factors of a variety of pathogens that infect both humans and animals. |

Reflecting their support of knowledge re-use and automated reasoning, ontologies have been implemented in a variety of applications for the enhancement of patient diagnosis, care management and clinical decision support [61-63]. A brief overview and further references are provided in [64]. In the fields of infectious disease, DSSs are commonly used in diagnostic assistance, guidance in the prescription of anti-infectives, biosurveillance, and vector control.

Some examples are provided in **Table S6**.

**Table S6 | IDO based DSSs**

| Antibiotic decision support systems (ADSSs) | Use of ADSSs has been shown to be effective in mitigating inappropriate antibiotic prescribing and lowering local antimicrobial resistance [65, 66]. To facilitate interoperability and widespread circulation of future ADSSs, a Bacterial Clinical Infectious Disease Ontology (BCIDO) has been developed from IDO Core [47]. |
| --- | --- |
| IDDAP [68] | IDDAP is a recently developed ontology-driven clinical decision support system for infectious disease diagnosis and antibiotic prescription. IDDAP makes use of an infectious disease diagnosis ontology that builds upon IDO Core. |
| Dengue Decision Support System (DDSS) [69, 70] | The DDSS is an ontology driven computational application developed at Colorado State University to guide the implementation of locally appropriate Dengue and Dengue Vector control programs. The DDSS is used in conjunction with Chaak, a cell phone-based system for i) the field capture of data relating to Dengue vector surveillance; and ii) the rapid transfer of the data to the central DDSS database [71]. |

**References**

1. Devare M, Aubert C, Laporte MA, Valette L, Arnaud E, Buttigieg PL. Data-driven agricultural research for development – a need for data harmonization via semantics. In: Jaiswal P, Hoehndorf R, editors. *Proceedings* *of the Joint International Conference on Biological Ontology and BioCreative (ICBO-BioCreative 2016)*. CEUR-WS.org; 2016. p. 33.
2. Hogan WR, Wagner MM, Brochhausen M, Levander J, Brown ST, Millet N. The Apollo Structured Vocabulary: an OWL2 ontology of phenomena in infectious disease epidemiology and population biology for use in epidemic simulation. *J Biomed Semant*. 2016; 7(50). doi:10.1186/s13326-016-0092-y.

3. Zheng J, Manduchi E, Stoeckert C. Development of an Application Ontology for Beta Cell Genomics Based on the Ontology for Biomedical Investigations. In: Dumontier M, Hoehndorf R, Baker CJO, editors. *Proceedings of the 4^th^ International Conference on Biomedical Ontology (ICBO 2013)*. CEUR-WS.org; 2013. p. 62-67.

4. Barton A, Rosier A, Burgun A, Ethier JF. The Cardiovascular disease ontology. In: Garbacz P, Kutz O, editors. *Formal Ontology in Information Systems: Proceedings of the 8th International Conference (FOIS 2014)*. Amsterdam: IOS Press; 2014. p. 409-414.

5. Coronavirus Infectious Disease Ontology. https://bioportal.bioontology.org/ontologies/CIDO. Accessed 27 Apr 2020.

6. Schriml LM, Arze C, Nadendla S, Chang YW, Mazaitis M, Felix V, et al. Disease ontology: a backbone for disease semantic integration. *Nucleic Acids Res*. 2012; 40:D940–D946. doi: 10.1093/nar/gkr972.

7. Hogan WR, Hanna J, Joseph E, Brochhausen M. Towards a Consistent and Scientifically Accurate Drug Ontology. In: Dumontier M, Hoehndorf R, Baker CJO, editors. *Proceedings of the 4^th^ International Conference on Biomedical Ontology (ICBO 2013)*. CEUR-WS.org; 2013. p. 68-73.

8. Zheng J, Cade JS, Brunk B, Roos DS, Stoeckert CJ, Sullivan SA, et al. Malaria study data integration and information retrieval based on OBO Foundry ontologies. In: Jaiswal P, Hoehndorf R, editors. *Proceedings* *of the Joint International Conference on Biological Ontology and BioCreative (ICBO BioCreative 2016)*. CEUR-WS.org; 2016. p. 38.

9. Influenza Ontology. https://bioportal.bioontology.org/ontologies/FLU. Accessed 27 Apr 2020.

10. Griffiths E, Dooley D, Graham M, Van Domselaar G, Brinkman FSL, Hsiao WWL. Context Is Everything: Harmonization of Critical Food Microbiology Descriptors and Metadata for Improved Food Safety and Surveillance. *Front Microbiol*. 2017; 8:1068. doi:10.3389/fmicb.2017.01068.

11. Hypertension Ontology. http://bioportal.bioontology.org/ontologies/HTN. Accessed 27 Apr 2020.

12. Cowell LG, Smith B. Infectious Diseases Ontology. In: Sintchenko V, editor. *Infectious Disease Informatics*. New York, NY: Springer; 2010. p. 373-95.

13. Brucellosis Ontology. https:// bioportal.bioontology.org/ontologies/IDOBRU. Accessed 27 Apr 2020.

14. Meningitis Ontology. https://github.com/cedricbere/IDOMEN. Accessed 27 Apr 2020.

15. Plant Disease Ontology. http://purl.obolibrary.org/obo/idoplant.owl. Accessed 27 Apr 2020.

16. *Staphylococcus aureus* Infectious Disease Ontology. https://github.com/awqbi/ido-staph. Accessed 27 Apr 2020.

17. Schistosomiasis Ontology. https://github.com/gaoussoucamara/idoschisto. Accessed 27 Apr 2020.

18. Hastings J, Ceusters W, Jensen M, Mulligan K, Smith B. Representing mental functioning: Ontologies for mental health and disease. *3rd International Conference on Biomedical Ontology (ICBO 2012)*. Citeseer; 2012. p. 1-5.

19. Ceusters W, Smith B. Foundations for a realist ontology of mental disease. *J Biomed Semant*. 2010; 1(10). doi:10.1186/2041-1480-1-10.

20. Mungall CJ, McMurry JA, Köhler S, Balhoff JP, Borromeo C, Brush M, et al. The Monarch Initiative: an integrative data and analytic platform connecting phenotypes to genotypes across species. *Nucleic Acids Res*. 2017; 45:D712–D722. doi:10.1093/nar/gkw1128.

21. Jensen M, Cox AP, Chaudhry N, Ng M, Sule D, Duncan W, et al. The neurological disease ontology. *J Biomed Semant*. 2013; 4(1):42. doi:10.1186/2041-1480-4-42.

22. He Y, Sarntivijai S, Lin Y, Xiang Z, Guo A, Zhang S, et al. OAE: The ontology of adverse events. *J Biomed Semant*. 2014; 5(29). doi:10.1186/2041-1480-5-29.

23. Bandrowski A, Brinkman R, Brochhausen M, Brush MH, Bug B, Chibucos MC, et al. The Ontology for Biomedical Investigations. *PLOS ONE*. 2016; 11(4):e0154556. doi:10.1371/journal.pone.0154556.

24. OBI-NIAID-GSC-BRC-view. https://bioportal.bioontology.org/ontologies/NIAID-GSC-BRC. Accessed 27 Apr 2020.

25. Brochhausen M, Zheng J, Birtwell D, Williams H, Masci AM, Ellis HJ, et al. OBIB-a novel ontology for biobanking. *J Biomed Semant*. 2016; 7(23). doi: 10.1186/s13326-016-0068-y.

26. Ray P, Diehl AD. The ocular disease ontology. In: Dumontier M, Hoehndorf R, Baker CJO, editors. *Proceedings of the 4^th^ International Conference on Biomedical Ontology (ICBO 2013)*. CEUR-WS.org; 2013. p. 119.

27. Schleyer TK, Ruttenberg A, Duncan W, Haendel M, Torniai C, Acharya A, et al. An ontology-based method for secondary use of electronic dental record data. *AMIA Jt Summits Transl Sci Proc*. 2013; p. 234–38.

28. Ontology of Host Pathogen Interactions. https://bioportal.bioontology.org/ontologies/OHPI. Accessed 27 Apr 2020.

29. Ontology of Laboratory Animal Medicine. https://bioportal.bioontology.org/ ontologies/OLAM. Accessed 27 Apr 2020.

30. Brochhausen M, Fransson MN, Kanaskar NV, Eriksson M, Merino-Martinez R, Hall RA, et al. Developing a semantically rich ontology for the biobank-administration domain. *J Biomed Semant*. 2013; 4(23). doi:10.1186/2041-1480-4-23.

31. Hicks A, Hanna J, Welch D, Brochhausen M, Hogan W. The ontology of medically related social entities: recent developments. *J Biomed Semant*. 2016; 7(47). doi:10.1186/s13326-016-0087-8.

32. Farinelli F, Almeida MB, Elkin PL, Smith B. OntONeo: The Obstetric and Neonatal Ontology. In: Jaiswal P, Hoehndorf R, editors. *Proceedings* *of the Joint International Conference on Biological Ontology and BioCreative (ICBO-BioCreative 2016)*. CEUR-WS.org; 2016. p. 35.

33. Ontology of Precision Medicine and Investigation https://bioportal.bioontology.org/ ontologies/OPMI. Accessed 27 Apr 2020.

34. Ethier JF, Barton A, Taseen R. An ontological analysis of drug prescriptions. *Applied Ontology* (2018) 13(4):273-294. doi:10.3233/ao-180202.

35. Planarian Phenotype Ontology. https://bioportal.bioontology.org/ontologies/PLANP. Accessed 27 Apr 2020.

36. He Y, Cowell LG, Diehl AD, Mobley H, Peters B, Ruttenberg A, et al. VO: Vaccine Ontology. In: Smith B, editor. *Proceedings of the 1st International Conference on Biomedical Ontology (ICBO 2009)*. Buffalo: NCOR; 2009. p. 172.

37. Goldfain A, Smith B, Arabandi S, Brochhausen M, Hogan W. Vital Sign Ontology. In: *Proceedings of the Workshop on Bio-Ontologies*, *ISMB*. 2011; p. 71-74.

38. Malaria Ontology. https:// github.com/VeuPathDB-ontology/IDOMAL. Accessed 27 Apr 2020.

39. Dengue Ontology. https://bioportal.bioontology.org/ontologies/IDODEN. Accessed 27 Apr 2020.

40. Sargeant D, Deverasetty S, Luo Y, Villahoz Baleta A, Zobrist S, Rathnayake V, et al. HIVToolbox, an integrated web application for investigating HIV. *PLOS ONE*. 2011; 6:e20122. doi: 10.1371/journal.pone.0020122.

41. Walls RL, Smith B, Elser J, Goldfain A, Stevenson DW, Jaiswal P. A plant disease extension of the infectious disease ontology. In: Cornet R, Stevens R, editors. *Proceedings of* *the 3^rd^ International Conference on Biomedical Ontology*. CEURS-WS.org; 2012. p. 1-5.

42. Ginsberg J, Mohebbi M, Patel R, Brammer L, Smolinski M, Brilliant L. Detecting Influenza Epidemics using Search Engine Query Data. *Nature*. 2009; 457(7232):2012-2014. doi:10.1038/nature07634.

43. Signorini A, Segre AM, Polgreen PM. The Use of Twitter to Track Levels of Disease Activity and Public Concern in the U.S. during the Inﬂuenza A H1N1 Pandemic. *PLOS ONE*. 2011; 6(5):e19467. doi:10.1371/journal.pone.0019467.

44. Generous N, Fairchild G, Deshpande A, Del Valle S, Priedhorsky R. Global disease monitoring and forecasting with Wikipedia. *PLOS Comput Biol*. 2014; 10(11):e1003892. doi: 10.1371/journal.pcbi.1003892.

45. Doan S, Ohno-Machado L, Collier N. Enhancing Twitter Data Analysis with Simple Semantic Filtering: Example in Tracking Influenza-Like Illness. In: Setti G, editor. *HISB’12: Proceedings of the 2012 IEEE Health Care Informatics, Imaging and Systems Biology*. Washington, DC: IEEE; 2012. p. 62-71. doi:10.1109/HISB.2012.21.

46. Magumba M, Nabende P, Mwebaze E. Ontology boosted deep learning for disease name extraction from Twitter messages. *Journal of Big Data*. 2018; 5(31). doi:10.1186/s40537-018 -0139-2.

47. Gordon CL, Pouch S, Cowell LG, Boland MR, Platt HL, Goldfain A, et al. Design and evaluation of a bacterial clinical infectious diseases ontology. *AMIA Annu Symp Proc*. 2013; p. 502– 511.

48. Lozano-Fuentes S, Bandyopadhyay A, Cowell LG, Goldfain A, Eisen L. Ontology for vector surveillance and management. *J Med Entom*. 2013; 50:1-14. doi:10.1603/me12169.

49. Matthews TC, Bristow FR, Grifﬁths EJ, Petkau A, Adam J, Dooley D, et al. The Integrated Rapid Infectious Disease Analysis (IRIDA) platform. *bioRxiv*. 2018; Available at: https://doi.org/10.1101/381830. Accessed 27 Apr 2020.

50. Xiang Z, Zheng W, He Y. BBP: Brucella genome annotation with literature mining and curation. *BMC Bioinform*. 2006; doi:10.1186/1471-2105-7-347.

51. Vaccine Investigation and Online Information Network. http://www.violinet.org/. Accessed 27 Apr 2020.

52. Cooper L, Meier A, Laporte MA, Elser J, Mungall CJ, Sinn BT, et al. The Planteome database: an integrated resource for reference ontologies, plant genomics and phenomics.

53. Eukaryotic Pathogen Genomics Database. https://eupathdb.org/eupathdb/. Accessed 27 Apr 2020.

54. VectorBase: Bioinformatics Resource for Invertebrate Vectors of Human Pathogens. http://vectorbase.org. Accessed 27 Apr 2020.

55. https://eurekalert.org/pub_releases/2019-10/uop-nat101519.php. Accessed 27 Apr 2020.

56. Zheng J, Cade JS, Brunk B, Roos DS, Stoeckert CJ, Sullivan SA, et al. Malaria study data integration and information retrieval based on OBO Foundry ontologies. In: Jaiswal P, Hoehndorf R, editors. *Proceedings* *of the Joint International Conference on Biological Ontology and BioCreative (ICBO-BioCreative 2016)*. CEUR-WS.org; 2016. p. 38.

57. Squires RB, Noronha J, Hunt V, García‐Sastre A, Macken C, Baumgarth N, et al. Influenza Research Database: An integrated bioinformatics resource for influenza virus research. *Influenza Other Respir Viruses*. 2012; 6(6): 404-416. doi:10.1111/j.1750-2659.2011.00331.x.

58. https://bioportal.bioontology.org/projects/IRD

59. Virus Pathogen Resource. http://www.viprbrc.org.

60. Sayers S, Li L, Ong E, Deng S, Fu G, Lin Y, et al. Victors: a web-based knowledge base of virulence factors in human and animal pathogens. *Nucleic Acid Res*. 2019; 47:D693-D700. doi:10.1093/nar/ gky999.

61. Zhang YF, Gou L, Zhou TS, Lin DN, Zheng J, Li Y, et al. An ontology-based approach to patient follow-up assessment for continuous and personalized chronic disease management. *J Biomed Inform*. 2017; 72:45–59. doi: 10.1016/j.jbi.2017.06.021.

62. Abidi S. A knowledge-modeling approach to integrate multiple clinical practice guidelines to provide evidence-based clinical decision support for managing comorbid conditions. *J Med Syst*. 2017; 41(12):193. doi:10.1007/s10916-017-0841-1.

63. Lin Y, Staes CJ, Shields DE, Kandula V, Welch BM, Kawamoto K. Design, development, and initial evaluation of a terminology for clinical decision support and electronic clinical quality measurement. *AMIA Annu Symp Proc*. 2015; p. 843–51.

64. Haendel MA, McMurry JA, Relevo R, Mungall CJ, Robinson PN, Chute CG. A Census of Disease Ontologies. *Annu Rev of Biomed Data Sci*. 2018; 1(1):305-331.

65. Thursky KA, Mahemoff M. User-centered design techniques for a computerized antibiotic decision support system in an intensive care unit. *Int J Med Inform*. 2007; 76:760-8.

66. Paterson DL. The role of antimicrobial management programs in optimizing antibiotic prescribing within hospitals. *Clin Infect Dis*. 2006; 42 Suppl 2: S90-5. doi:10.1086/499407.

68. Shen Y, Yuan K, Chen D, Colloc J, Yang M, Li Y, et al. An ontology-driven clinical decision support system (IDDAP) for infectious disease diagnosis and antibiotic prescription. *Artif Intell Med*. 2018; 86:20–32.

69. Eisen L, Coleman M, Lozano–Fuentes S, McEachen N, Orlans M, Coleman M. Multi-disease data management system platform for vector-borne diseases. *PLOS Negl Trop Dis*. 2011; 5:e1016. doi:10.1371/journal.pntd.0001016.

70. Lozano-Fuentes S, Barker CM, Coleman M, Park BB, Reisen WK, Eisen L. Emerging information technologies to provide improved decision support for surveillance, prevention, and control of vector-borne diseases. In: Jao C, editor. *Efficient Decision Support Systems: Practice and Challenges in Biomedical Related Domain*. Rijeka, Croatia: InTech-Open Access Publisher; 2011. p. 89-114.

71. Lozano-Fuentes S, Wedyan F, Hernandez-Garcia E, Devadatta S, Ghosh S, Bieman JM, et al. Cell phone-based system (Chaak) for surveillance of immatures of dengue virus mosquito vectors. *J Med Entomol*. 2013; 50(4):879-89. doi:10.1603/me13008.
