# Supplementary material for: The Infectious Disease Ontology in the age of COVID-19
Source: J Biomed Semantics. 2021 Jul 18;12:13. doi: 10.1186/s13326-021-00245-1 (PMC8286442; doi:10.1186/s13326-021-00245-1)
Supplement: Supplementary file 2 — Additional file 2. The Infectious Disease Ontology Extensions: Some Issues. (.docx). Several IDO ontologies require significant reengineering if they are to be considered bona fide extensions of IDO Core. This document provides an overview of some issues concerning specific IDO extensions, while providing some suggestions for how they can be addressed. [file 13326_2021_245_MOESM2_ESM.docx]

**The Infectious Disease Ontology Extensions: Some Issues**

**Shane Babcock and John Beverley**

As we move forward in our efforts to refine the IDO suite, many of the IDO extensions will require reengineering. Here we detail some of the issues that need to be addressed within specific IDO extensions.

I. *The Coronavirus Infectious Disease Ontology (CIDO)*

Alignment with IDO Core will require much curation. For example, in CIDO:

Created terms do not have textual definitions

There are two distinct hierarchies, one for *entity* and one NCBITaxon:*taxonomic rank*

Within the entity subclass BFO:*continuant*:

ChEBI:*role* is as a sibling class of BFO:*role*, and distinct definitions are provided

OBI:*host role* is a sibling class of IDO:*host role*

OGMS:*disease* is a sibling class of NDF-RT:*Diseases, Manifestations, or Physiological States*

Related, the imported class IDO:*infectious disorder* has no child terms, despite coronavirus infections being in purview of CIDO

Moreover, CIDO has not introduced relevant subclasses of IDO:*host* such as IDO:*pathogen host* or IDO:*intermediate host*, presumably needed to track the spread of coronaviruses

IDO:*pathogen* has only the subclass IDO:*infectious agent*, though further specification is expected in CIDO given its focus on viruses

ChEBI:*antiviral agent* is imported as a BFO:*role* but there is no corresponding material entity bearer; similarly for ChEBI:*antiviral drug*; CIDO does import VO:*viral vaccine* but vaccines are not antivirals

ChEBI:*molecular entity* is defined as a direct subclass of both BFO:*material entity* and BFO:*object*

CIDO inherits from UBERON the class *anatomical entity* as a subclass of BFO:*material entity* and yet is defined as either a material or immaterial entity; this is reflected in CIDO also importing under BFO:*material entity* the UBERON class *material anatomical entity*. BFO:*material entity* is disjoint from BFO:*immaterial entity*, though material entities may have immaterial parts

Turning to the entity subclass BFO:*occurrent*:

CIDO only imports BFO:*process* as a child class of BFO:*occurrent,* and so lacks the resources to specify temporal intervals and points of time, which thereby undermines CIDO’s ability to represent the spread of coronaviruses over time; this suggests CIDO is unable to represent antivirals specifically targeting process parts of coronavirus reproduction, as discussed in the main document

In particular, CIDO cannot obviously at this point represent the coronavirus reproduction cycle

Moreover, OGMS:*disease course* has only the subclass IDO:*infectious disease course*, lacking further expected specification for coronaviruses

CIDO does, however, create terms for coronavirus, e.g. CIDO:*coronavirus infectious disease process* and sibling subclasses such as CIDO: *SARS-CoV disease process* and CIDO:*Human coronavirus 229E DP* - all processes – some of which are related to the other coronavirus terms that have been imported from, say DOID, and other ontologies, but not all

Turning lastly to object properties:

CIDO’s relation CIDO:*is host of* is not defined

Concerning this last point, IDO Core provides resources that might be useful for the construction of the needed definition. CIDO imports IDO:*host* and IDO:*infectious agent*, and could also import IDO:*infectious structure*, presumably the relata of this relation. Recall that IDO Core defines a host as an organism having some material entity as part or as part of its extended organism. As a first pass, one might define:

- *is host of* =_def_ host x is part of extended organism z and z has proper part material entity y and x≠y

CIDO might then sharpen this to a sub-relation holding between a host and a *virus* that is an *infectious structure*, which makes sense given the domain of interest:

- *is host of virus* =_def_ host x is host of virus y and y is an infectious structure

That said, this relation is of limited use, since it would be false to assert anything stronger than some host is the host of some virus infectious agent. In particular, it would be false to assert that all hosts are hosts of some virus infectious agent. We might define instead for CIDO the following variation, relying on the BFO:*part of* relation:

- *virus has host* =_def_ x is a virus and x is an infectious structure and has established an infection in proper part host y of extended organism z and x≠y

Which allows one to make the true claim that all virus infectious structures that have established an infection have some host.

CIDO developers intend to align with IDO and relevant, related, ontologies, e.g. OGMS, BFO. Presently, CIDO and IDO developers are working to ensure alignment.

II. *Brucellosis Ontology* (*IDOBRU*)

Though IDOBRU is largely in alignment with IDO Core and other relevant ontologies, there are some aspects of the ontology needing to be addressed. We have begun to address some of these issues. For example:

- IDOBRU previously contained the term *brucellosis disposition*, redundantly defined as “A brucellosis disposition that is the disposition to be transmitted from an infected non-human host to a human host”, while its parent IDO:*zoonotic disposition* is defined as “the disposition to be transmitted from an infected non-human host to a human host”. IDOBRU:*brucellosis disposition* should not be a child of IDO:*zoonotic disposition*, given that *Brucella* can be transmitted from humans to humans in addition to being transmitted zoonotically. To fix this issue, we replaced *brucellosis disposition* with the new term *Brucella infectious disposition*, which we define as follows:

*Brucella infectious disposition* =def An infectious disposition that is the disposition of Brucella to be transmitted to a host and establish a Brucella infectious disorder.

Since a Brucella infectious disorder is the material basis of the brucellosis disease, this definition captures the fact that Brucella is a cause of brucellosis. For zoonotic transmission, we may also define a new term, *brucella zoonotic disposition*, along the following lines:

*Brucella zoonotic disposition* =def An infectious disposition that is the disposition of Brucella to be transmitted from an infected non-human host to a human host.

Several textual definitions are circular:

- IDOBRU:*brucellosis pathogen role* is a subclass of IDO:*pathogen role*, but the former was previously defined circularly as “a pathogen role of being a brucellosis pathogen”. We have revised this definition by refinement of the differentia used to define IDO:*pathogen role*, replacing the term *pathogenic disposition* with *Brucella infectious disposition,* as follows:

*brucellosis pathogen role* =_def_ A pathogen role borne by Brucella bacterium when contained in a host in which its Brucella infectious disposition can be realized.

Other IDO extensions could provide similar definitions for pathogen roles corresponding to *Staphylococcus aureus*, the influenza viruses, malaria parasites, and so on.

- IDOBRU:*veterinarian role* is also circularly defined as “a role of being a veterinarian”, and similar remarks apply to IDOBRU:*animal dealer role*, IDOBRU:*bioterrorism agent role,* IDOBRU:*butcher role*, and several others
- IDOBRU:*drinking function* is similarly circularly defined as “the drinking function of realizing a drinking disposition”

Some classes have lone children, such as:

- IDOBRU:*process of establishing* *Brucella infection in host* is the sole child of IDO*:process of establishing an infection*

Of note, IDO Core developers have begun working with IDOBRU developers to align the epidemiology related content of the two ontologies. As noted in the main text, IDO Core contains qualities of disease affected populations, such as infectious disease incidence rate, infectious disease mortality rate, and infectious disease endemicity. We have recently added terms for the corresponding sites at which these qualities are instantiated. For example: infectious disease endemic site, infectious disease free site, and infectious disease non-endemic site. Adding these classes allows them to serve as parent classes for the brucellosis specific classes brucellosis endemic site, brucellosis free site, and brucellosis non-endemic site, currently used in IDOBRU.

III. *Influenza Ontology* (*FLU*)

As there are no previous publications in which FLU has been described we discuss its content in some detail, as well as our recent efforts to achieve alignment with IDO Core. In the most recent published version of FLU^[[1]](#footnote-1)^ (dated Aug 20, 2015), classes are currently divided between two separate *is_a* hierarchies. One of these is built upon the outdated BFO version 1.1. The other is built upon BFO version 2.0. For the purposes of our exposition, we refer to these hierarchies as FLU1.1 and FLU2.0 respectively.

Below is a summary of some of the FLU terms appearing within FLU1.1, organized according to the BFO version 1.1 classes under which they fall:

- FLU terms descending from *material entity* include: terms related to influenza surveillance such as *bird identification band*; terms for influenza proteins such as *influenza hemagglutinin* and *influenza neuraminidase*; terms relating to influenza prevention and treatment such as *organism treated with antibiotics*, *organism treated with antiviral drug*, and *vaccinated organism*
- Subclasses of OBI:*organism* such as *Influenza virus A*, *Influenza virus B*, and *Influenza virus C*
- A variety of terms for influenza host organisms descending from NCBITaxon:*Eukaryota*.
- FLU terms descending from *role* include a variety of terms relating to influenza surveillance and sequencing such as *extractor role*, *sequence annotator role*, *specimen collector role* (which appear as direct children of OBI:*investigation agent role*)
- Descending from *quality* is the FLU term *life stage*, which has several children including *fledgling*, *hatch year*, *nestling* and *yearling* (for the representation of life stages of avian influenza hosts)

In future iterations of FLU, FLU1.1 terms and children will be reassigned to the corresponding BFO version 2.0 classes. Notable challenges include:

- FLU1.1’s *planned process* hierarchy is identical to that of FLU2.0, except in the former OBI:*planned process* descends from the outdated BFO1.1 term *processual entity*
- FLU1.1 OBI:*planned process* has sibling classes that do not appear in FLU2.0, such as FLU:*enrichment process*, OGMS:*health care process*, and OGMS:*treatment*.
- In both *planned process* hierarchies of FLU2.0 and FLU1.1, the OBI:*collecting specimen from organism* and OBI:*environmental material collection* are children of FLU:*specimen creation*, defined as “a planned process with the objective of obtaining specimen.” However, in FLU1.1, but not FLU2.0, the same OBI classes also appear as children of the functionally equivalent FLU:*specimen collection*, which is defined as “the process of collecting a specimen, either from an organism or from an environment”

This is list is incomplete and is just a snapshot of the terms that require reassignment.

Below is a summary of some of the FLU terms appearing within FLU2.0. All of those that were newly created for FLU descend either from IAO:*information content entity* (under BFO:*generically dependent continuant*) or from OBI:*planned process* (under BFO:*process*):

- Under the IAO:*information content entity* subclass IAO:*data item* there are a variety of FLU terms representing data items pertaining to influenza surveillance and influenza virus sequences. Subclasses of IAO:*data item* include *data about a collection location*, *data about a genbank entry*, *nucleic acid sequence*, and *influenza virus antigenic data*
- IAO:*scalar measurement datum* has as subclasses FLU terms such as *hemagglutinin titer*, *sequence coverage*, *initial oxygen saturation* and *virus pathogenicity after passage*
- IAO:*plan specification* includes a variety of FLU terms pertaining to influenza surveillance and virus sequencing such as *virus isolation protocol*, *sequencing protocol*, and *specimen collection protocol*
- FLU imports the IAO terms *material information bearer*, *photographic print*, *information carrier*, and *author role* (the first two as children of BFO:*material entity*, the third as a child of BFO:*quality* and the fourth as a child of BFO:*role*)
- OBI:*planned process* subclass OBI:*assay* has as children FLU terms including *hemagglutinin typing* and *neuraminidase typing*. Both of have the child OBI:*viral hemagglutination inhibition assay*
- OBI:*assay* subclass OBI:*sequencing assay* has the FLU term *genomic sequencing* as a child
- Direct or indirect FLU subclasses of OBI:*material component separation* include *virus amplification*, *virus passage* and *virus isolation*
- FLU:*virus isolation* is a child of OBI:*extraction* (itself a child of OBI:*material component separation*).

While obviously relevant to the influenza domain, many of the data related FLU terms listed above apply to viral diseases more generally. Likewise, the FLU processual entities *genomic sequencing*, *virus amplification*, *virus passage*, *virus isolation*, and *antiviral treatment* are not specific to the influenza domain.

FLU also has some issues with regard to what is defined and what is expected to be defined but not. For example:

- FLU:*organism treated with antiviral drug* is defined as “an organism that has been treated with an antiviral drug.” But there is no defined relation *treated by*, and the use of “drug” is ambiguous, i.e. between meaning a material entity, or meaning a role of some material entity, the latter used by ChEBI from which FLU imports several terms
- FLU:*antiviral drug*^[[2]](#footnote-2)^ has one child, FLU: *neuraminidase inhibitor drug* defined as “A drug targeted at the influenza virus, which works by blocking the function of the viral neuraminidase protein, thus preventing the virus from reproducing by budding from the host cell.” IDO Core recommends explicating phenomena used in the definition in part as involving dispositions. Yet, *antiviral disposition* is not currently present within FLU
- IDO:*antibacterial* is defined in terms of the class IDO: *antibacterial disposition*, but this class has not been included in FLU
- More generally, FLU includes only one disposition: FLU:*hemagglutination disposition* defined as the disposition to initiate hemagglutination processes, yet introduces a class FLU:*organism treated with antiviral drug* simply defined as an organism that has been treated by an antiviral drug; IDO Core terms like *protective resistance*, to characterize flu strain resistance to antiviral drug treatments would surely be useful here

We are currently developing an updated version of FLU, which can be found here: https://github.comPhiBabs935/IDO-FLU. As part of this project, we have reassigned the following FLU terms to VIDO: *virus isolation protocol*, *virus amplification*, *amplified virus preparation*, *virus passage*, *virus passage date*, *virus pathogenicity after passage*, *virus isolation*, *antiviral treatment*, and *antiviral treatment date*. After searching ONTOBEE, we found that a variety of OBO Foundry and Library ontologies contained existing terms that were suitable to replace FLU terms that were out of scope. These ontologies, to name a few, include the Ontology of Biomedical Investigations (OBI)^[[3]](#footnote-3)^, the VEuPathDB Ontology (EUPATH), the Genomic Epidemiology Ontology (GenEpio), and the Vaccine Ontology (VO). To give just a few examples of the relevant term replacements: FLU:*specimen collection*, FLU:*sequencing protocol*, FLU:*sequence coverage*, and FLU:*vaccinated organism* have been replaced, respectively, with OBI:*specimen collection process*, EUPATH:*sequencing protocol*, GenEpio:*read coverage*, and VO:*vaccinated organism*. In our updated version of FLU, all obsoleted FLU terms can be found under *Obsolete Class* with annotations pointing to the higher-level ontology term with which they have been replaced.

Where suitable replacements could not be found, we have made requests to have various FLU terms ported out to more appropriate upper-level OBO Foundry ontologies. First instance, we have suggested exporting a number of terms to both OBI and GenEpio. Thus, see the following issue trackers: https://github.com/obi-ontology/obi/issues/1286; and: https://github.com/ GenEpiO/genepio/issues/22. Upon request, the term *RNA extraction protocol* was added to the Experimental Factor Ontology (EFO), and then imported to replace FLU:*RNA extraction protocol* (see: https://github.com/EBISPOT/efo/issues/940). Related, we have imported a number of existing EFO terms to replace out of scope FLU terms (e.g., FLU:*extraction protocol* has been replaced by an equivalently labeled term from EFO.)

We have also created many new influenza related terms for FLU and have also imported many new terms to cover facets of the domain not covered in previous versions of the ontology. In doing so we have reengineered FLU as a direct extension of VIDO. Many of the new terms that were created for FLU mirrored the creation of comparable terms for IDO-COVID-19.

IV. *The HIV Ontology* (*HIV*)

HIV imports neither BFO nor IDO Core; HIV terms are not organized into a hierarchy, and the majority are direct children of OWL:*thing*, i.e. the ontology is primarily a flat list of terms having few child classes. If it is to be considered an extension of IDO Core, the HIV ontology will need significant updating and re-organization of its content. Indeed, it is difficult to make suggestions for alignment with IDO Core, without the presence of any hierarchy characteristic of ontologies.

While the ontology only focuses on HIV virus biology and genetics, with a few terms pertaining to antiviral drugs and resistance. There is no coverage of terms pertaining to transmission or pathogen hosts.

V. *The Malaria Ontology* (*IDOMAL*) *and The Dengue Ontology* (*IDODEN*)

As noted in the main text, IDOMAL has recently been obsoleted. It will eventually be replaced with a new IDO extension for malaria. That said, we believe that careful consideration of IDOMAL’s errors will be instructive for when we begin to develop its future replacement. In the following, for the sake of brevity we will refer to this future replacement as MAL 2.0. While IDOMAL terms will not be reused, there is still much useful information represented in the ontology that can be used as a loose template for constructing MAL 2.0.

As IDOMAL and IDODEN were built according to the same architecture, they share the same alignments and discrepancies with IDO Core. Thus, we consider the two ontologies together. Each is a well-organized hierarchy, but that hierarchy is built on BFO1.1 rather than BFO2.0. Moreover, though many key terms are imported from IDO Core, neither inherit IDO Core’s hierarchical structure. Alignment with IDO Core clearly requires importing relevant terms consistent with IDO Core’s hierarchy.

One persistent problem worth discussion is the conflation of entity types. In some cases, *qualities* and *realizable entities* are conflated with *occurrent* entities, and in other cases *dispositions* are conflated with *qualities*. For example, in both ontologies:

- *symptom* is a subclass of *condition*, itself a child of BFO:*realizable entity*. But several subclasses of *symptom* are processual entities, conflating processes and realizable entities
- RO:*happens_during*, which is a relation between processes, is often asserted as holding between continuants, e.g. IDOMAL:*circulatory collapse* is a subclass of *symptom* and *happens_during* some *clinical manifestation of falciparum malaria*, but the latter is a subclass of *quality of malaria;* IDODEN:*ascites* is a subclass of *symptom* and *happens_during* some *clinical manifestation of dengue*, but the latter is a subclass of *quality of dengue fever*
- BFO:*continuant entity* is conflated with BFO:*occurrent entity*, e.g. IDOMAL:*clinical manifestation of malaria* is a subclass of *part_of* some *progression of malaria* and IDODEN:*clinical manifestation of dengue* is asserted as a *progression of dengue fever*, so in each case a *quality* is asserted as a part of some *process*, but BFO:*continuant entity* can be a part of some BFO:*occurrent entity*
- *contagiousness* is defined as an ability to spread from one person or species to another, and yet IDODEN follows IDO Core in classifying *contagiousness* as a disposition, while IDOMAL classifies it as a *quality of malaria*
- *zoonotic*, defined as “a disease that can be transmitted from animals to people”, is classified in IDOMAL as a *quality of malaria*, and in IDODEN as a *quality of dengue fever*

The last two examples highlight a major issue arising from the reliance on placeholder classes like *quality of malaria* and *quality dengue fever*, defined, respectively, as “A quality that refers to malaria” and as “A quality that refers to dengue fever”. Such placeholder classes often result in the inheritance of properties that do not hold for a type. Thus, while *contagiousness* is a subtype of *quality of malaria* in IDOMAL, not all instances of *contagiousness* refer to malaria. The same point applies to the classification of *zoonotic* as a *quality of malaria* in IDOMAL and its classification as *quality of dengue fever* in IDODEN. Similarly, in both IDOMAL and IDODEN, *immunity* is classified as a *quality of host*, which is defined as “A quality which refers to the host”. But not all instances of immunity refer to a host, as an organism can have an immunity without being a host. Or consider the class *quality of vector*, defined as “A quality which refers to vectors.” In both IDOMAL and IDODEN there are many classes, including *flight capacity, male fecundity*, and *zoophily*, which are classified as subtypes of *quality of vector* even though instances of these types can be, and are, present in insects that do not happen to be vectors.

IDOMAL does contain many terms, for which newly created variants should be added to MAL 2.0. But the introduction of such terms will require the correction of some issues. For example:

- IDOMAL:*antiparasitic chemical compound* is defined as “A chemical compound bearing an antiparasitic disposition” and includes comprehensive subclasses, but since IDOMAL does not import IDO, this is not a subclass of IDO:*antiparasitic*, but rather of ChEBI:*chemical compound*. This issue of course can easily be fixed by introducing IDO:*antiparasitic* into MAL 2.0 and populating this class with new replacements for IDOMAL:*antiparasitic chemical compound* and its subclasses. Likewise, for similar cases.
- IDOMAL:*malaria vector* is a subclass of *host role* defined as “An anapheline mosquito transmitting malaria”. Strictly speaking, a malaria vector is a transporter of the pathogens of malaria. For MAL 2.0, malaria pathogen-specific child terms are needed for *infectious agent vector role*.

The same can be said for many classes descending from IDOMAL’s problematic placeholder classes. In MAL 2.0 replacement terms for these classes will be created and be reassigned, where possible, to hierarchies descending from appropriate upper-level terms from IDO Core. Where this is not possible, the IDO Core developers will discuss the possibility of adding new classes to IDO Core.

Recently, IDOMAL was inherited by the Stoeckert team at the University of Pennsylvania, which is hosting the ontology for legacy purposes here: <https://github.com/VeuPathDB-ontology/IDOMAL>. As part of a recently funded initiative to revamp and integrate the Eukaryotic Pathogen Genomics Database (<https://eupathdb.org/eupathdb/>) and the Bioinformatics Resource for Invertebrate Vectors of Human Pathogens (VectorBase: <http://vectorbase.org>), the Stoeckert team has replaced many terms with IDOMAL prefixes that were previously used to annotate data in VectorBase – which are not really infectious disease terms per se, and so should not have IDOMAL prefixes - with terms drawn from other OBO Foundry ontologies. Specifically:

IDOMAL:0000129 (Plasmodium falciparum), IDOMAL:0000131 (Plasmodium ovale), IDOMAL:0000132 (Plasmodium vivax), IDOMAL:0000133 (Plasmodium malariae), IDOMAL:0000222 (enzyme-linked immunosorbent assay), IDOMAL:0000443 (gravid), IDOMAL:0000552 (Giemsa staining), IDOMAL:0000653 (larva), IDOMAL:0000654 (pupa), IDOMAL:0000655 (adult), IDOMAL:0000658 (third instar larva), IDOMAL:0000659 (fourth instar larva), IDOMAL:0001254 (population).

They are now using NCBITaxon for the species, OBI for the assay related terms and for *population*, and UBERON for the life stages.

Of note, to represent the varying degrees to which diseases can be endemic within different populations, we have introduced to IDO Core the following epidemiology terms which were originally created for IDOMAL: holoendemicity, hypoendemicity, and mesoendemicity.

VI. *The Schistosomiasis Ontology* (*IDOSCHISTO*)

IDOSCHISTO will require significant rebuilding if it is to be considered a bona fide extension of IDO Core. IDO CORE is imported in full, and certain key classes descend from IDO Core classes (e.g. *Schistosoma* *is_a* IDO:*infectious agent*, *Schistosomiasis* *is_a* IDO:*infectious disease*). Still, most IDOSCHISTO terms lack textual definitions, and many other classes fall outside the BFO entity hierarchy entirely. Rather, they appear as subclasses of classes that are themselves direct children of OWL:*thing*. For example:

- *sample_for_direct _diagnosis*
- *snail_survival_condition*
- *schistosoma_survival_condition*
- *schistosomiasis_pathological_process*

While the ontology’s developers acknowledge the importance of reusing terms from established OBO Foundry ontologies, in various cases IDOSCHISTO fails to adhere to the principle of orthogonality. For example:

- IDOSCHISTO:*drug* and IDOSCHISTO*:vaccine* were newly created instead of reusing CHEBI:*drug* and VO:*vaccine*

Dr. Camara has recently informed us (personal communication) that he and his team hope to soon begin a new project studying schistosomiasis and fasciolosis in Senegal. In coordination with this project they have plans to revise and update IDOSCHISTO. We will be in communication with their team to help ensure consistency and IDO Core conformity.

VII. *The Meningitis Ontology* (*IDOMEN*)

IDOMEN, in its current iteration, suffers from several issues, and is far from alignment with BFO, OGMS, and IDO Core despite importing each in full. Of importance:

- IDOMEN is exceedingly difficult to navigate, due to the redundant assertion of classes
- IDOMEN’s continuant classes all appear as direct children of BFO:*continuant* (the same can be said for its occurrent hierarchy)
- IDOMEN sometimes fails to adhere to the principle of orthogonality, for example IDOMEN adds the new terms *patient*, *patient role*, and *vaccination* to serve, respectively, as the parents of the IDOMEN terms *meningitis patient*, *meningitis patient*, and *vaccination campaign* rather than import OAE:*patient*, OBI:*patient role*, and VO:*vaccination*. Similarly, to represent meningitis epidemic risk factors, IDOMEN adds a number of new terms for environmental factors, including terms for climatic factors. IDOMEN should consider reusing terms from ENVO
- Many IDOMEN terms lack textual definitions
- IDO:*infectious disposition* has the sole child IDOMEN:*meningitis disposition*

VII. *The importation of disease terms from the Human Disease Ontology (DOID)*

While some IDO extensions import disease terms from DOID we do not recommend this in all cases moving forward. DOID purports to follow OGMS. While many DOID disease terms are defined in terms of underlying disorders, this is not so in all cases. In general, DOID disease terms often contain more information than is appropriate. Consider for instance DOID: *influenza*:

*influenza* =def. A viral infectious disease that results in infection, located in respiratory tract, has_material_basis_in Influenzavirus A, has_material_basis_in Influenzavirus B, or has_material_basis_in Influenzavirus C, which are transmitted by droplet spread of oronasal secretions during coughing, sneezing, or talking from an infected person. It is a highly contagious disease that affects birds and mammals and has symptom chills, has symptom fever, has symptom sore throat, has symptom runny nose, has symptom muscle pains, has symptom severe headache, has symptom cough, and has symptom weakness.

A good definition captures just those essential features of the class, including its genus, as well as the differentia that distinguishes it from other subtypes of that genus. Most of the features included in the above definition are not differentia. Location in the respiratory tract, transmissibility through droplet spread, and many of the mentioned symptoms, are features common to a variety of viral infectious diseases. Such information would be better relegated to the term’s associated OWL axioms, or an editor’s comment. Notice the definition also gets things backwards by saying that *influenza* results in an infection. Rather, the infection is the basis of the disease. A better, OGMS-based template is provided by IDOSA: *Staphylococcus aureus infectious disease*:

*Staphylococcus aureus infectious disease* =def. Infectious disease that has a staphylococcus aureus infectious disorder as its material basis.

Accordingly, *influenza* ought to be defined as follows:

*influenza* =def. Viral infectious disease that has as its material basis either an Influenzavirus A infectious disorder, an Influenzavirus B infectious disorder, or an Influenzavirus C infectious disorder.

1. https://bioportal.bioontology.org/ontologies/FLU/ [↑](#footnote-ref-1)
2. Note, as the term FLU:*antiviral drug* has applicability to viral infections in general, in our developmental version of FLU we have replaced this term with ChEBI:*antiviral drug*. [↑](#footnote-ref-2)
3. The majority of existing replacement terms were taken from OBI. This is not a surprise, as a variety of persons associated with the development of OBI helped develop FLU. It appears that many of the terms that were created for FLU very early on – prior to OBI’s creation – were succeeded by more or less functionally equivalent OBI terms. [↑](#footnote-ref-3)
