# Supplementary material for: The Infectious Disease Ontology in the age of COVID-19
Source: J Biomed Semantics. 2021 Jul 18;12:13. doi: 10.1186/s13326-021-00245-1 (PMC8286442; doi:10.1186/s13326-021-00245-1)
Supplement: Supplementary file 3 — Additional file 3: Case Study. IDOSA and methicillin resistant Staphylococcus aureus (.docx) [file 13326_2021_245_MOESM3_ESM.docx]

**Additional File 3**

**Case Study: IDOSA and methicillin resistant Staphylococcus aureus**

IDO:*protective resistance* is used to model the resistance of certain bacteria to antibiotic drugs. For this purpose, the following subtypes of *protective resistance* are asserted in IDO Core:

*drug resistance* =def. Protective resistance that mitigates the damaging effects of a drug.
*antibiotic resistance* =def. Drug resistance that mitigates the damaging effects of an antibiotic.

Beta-lactam antibiotics such as methicillin are the most widely used antibiotics, and most work by preventing bacterial cell wall construction. They act by binding to and inhibiting the penicillin-binding-proteins (PBPs) within bacteria that facilitate the synthesis of peptidoglycan molecules, thus compromising the structural integrity of the cell wall. In response to the widespread use of beta-lactam antibiotics, some bacteria have rapidly evolved novel-structured PBPs which lack an affinity for these antibiotics, thus rendering them less effective. In IDO we aim to provide an ontological representation of resistance that reveals the active mechanisms that produce resistance—in the case of resistant bacteria, the active dispositions inhering in novel-structured PBPs that inhibit antibiotics from manifesting their damaging effects [1].

Consider the case of *methicillin resistant Staphylococcus aureus* (MRSa). MRSa’s resistance to methicillin is conferred by PBP2a, a PBP that lacks affinity for methicillin and is the product of the gene *mecA*. The need to provide a coherent and consistent understanding of the mechanisms underlying MRSa antibiotic resistance is one impetus for the development of the *Staphylococcus aureus* Infectious Disease Ontology (IDOSA), an extension of IDO covering entities specific to Staph aureus (Sa) infectious diseases [1, 2].

IDOSA’s main hierarchy is built on BFO, and imports IDO Core in full. IDOSA provides terms covering all entities relevant to antibacterial resistance in Sa, including terms for Sa proteins (from the Protein Ontology [3]), terms for genes and gene products, (from the Sequence Ontology [4]) terms for biological processes (from the GO Biological Process Ontology), terms for anatomical sites of infection (from the UBERON anatomy ontology [5]), and terms for antibiotics (from Chemical Entities of Biological Interest (ChEBI) [6]).

IDOSA imports the term *staphylococcus aureus* from NCBITaxon, while adding the following subclasses:

*methicillin-resistant Staphylococcus aureus* =def. Organism of type Staphylococcus aureus that has resistance to beta-lactam antibiotics.

*methicillin-susceptible Staphylococcus aureus* =def. Organism of type Staphylococcus aureus that is susceptible to beta-lactam antibiotics.

Both are defined in terms of IDOSA *resistance to beta-lactam antibiotic*, which itself is a subclass of IDO:*antibiotic resistance* and defined as follows:

*resistance to beta-lactam antibiotic* =def. Antibiotic resistance that mitigates the damaging effects of a beta-lactam antibiotic.

With these terms and definitions, we can characterize both *Methicillin-susceptible Staphylococcus aureus*’s (MSSa) susceptibility, and MRSa’s resistance, to beta-lactam antibiotics in terms of *protective resistance* and blocking dispositions [1]).

MSSa is susceptible to the damaging effects of methicillin because it *lacks* *protective resistance* to that drug. Characterized positively, MSSa’s PBPs have the disposition to undergo a methicillin PBP binding process that *negatively_ regulates* the synthesis of peptidoglycan, thereby interfering with the formation of a stable cell wall. Affinity for methicillin thus acts as a blocking disposition for the PBPs’ disposition to synthesize peptidoglycan.

In the case of MRSa, in contrast, the disposition of its PBP2a parts to synthesize peptidoglycan, and thereby participate in the construction of a stable cell wall (which *negatively_regulates* methicillin binding), cannot be blocked. Thus, MRSa’s protective *antibiotic resistance* to methicillin can be seen as an active response in which PBP2a manifests a disposition to mitigate the damaging effects of methicillin. [2] shows how the formal representation of these relations can be used in association with instance data to draw inferences that may facilitate automated drug discovery and guide treatment decisions in specific types of cases.

The IDO Core account of protective resistance can be applied also to other cases, such as the resistance against HIV-1 conferred by CCR5-Δ32, and the resistance against malaria conferred by the sickle cell trait [1]. CCR5-Δ32 is a deletion mutation of the CCR5 gene resulting in cells which lack a functioning CCR5 receptor on their surfaces. In this case, the disposition of individuals with the CCR5-Δ32 mutation to develop cells that lack CCR5 on their surface acts as a blocking disposition for the disposition of HIV-1 to bind to a CCR5 molecule. *Plasmodium falciparum*, one of the infectious agents that causes malaria, has a disposition to spread through the host by replicating within the host’s red blood cells, lysing from those cells, and then entering again into further red blood cells—a process that is reduced in dense, dehydrated red blood cells. In individuals with the sickle cell hemoglobin gene, red blood cells have a disposition to become dehydrated and thus increase in density. This disposition acts as a blocking disposition for the disposition of plasmodium to spread through red blood cells, a process requiring hydrated red blood cells.

**References**

1. Goldfain A, Smith B, Cowell LG. Towards an ontological representation of resistance: the case of MRSA. *J Biomed Inform*. 2011; 44:35-41. doi:10.1016/j.jbi.2010.02.008.

2. Goldfain A, Smith B, Cowell LG. Constructing a lattice of infectious disease ontologies from a staphylococcus aureus isolate repository. In: Cornet R, Stevens R, editors. *Proceedings of* *the 3^rd^ International Conference on Biomedical Ontology (ICBO 2012)*. CEURS-WS.org; 2012. P. 1-5.

3. Natale DA, Arighi CN, Blake JA, Bona J, Chen C, Chen S, et al. Protein Ontology (PRO): enhancing and scaling up the representation of protein entities. *Nucleic Acids Res*. 2017; 45: D339-D346. Doi: 10.1093/nar/gkw1075.

4. Eilbeck K, Lewis SE, Mungall CJ, Yandell M, Stein L, Durbin R, et al. The Sequence Ontology: a tool for the unification of genome annotations. *Genome Biol*. 2005; 6(5):R44. doi: 10.1186/gb-2005-6-5-r44.

5. Haendel MA, Balhoff JP, Bastian FB, et al. Unification of multi-species vertebrate anatomy ontologies for comparative biology in Uberon. *J Biomed Semant*. 2014; doi:10.1186/2041-1480-5-21.

6. Degtyarenko K, Matos P, Ennis M, et al. ChEBI: a database and ontology for chemical entities of biological interest. *Nucleic Acids Res*. 2008; 36:D344-D350. doi:10.1093/nar/ gkm791.
